# Full-Length Transcriptome Sequencing Combined with RNA-Seq to Analyze Genes Related to Terpenoid Biosynthesis in *Cinnamomum burmannii*

**DOI:** 10.3390/cimb44090288

**Published:** 2022-09-12

**Authors:** Siyuan Guo, Jiahao Liang, Zhiwei Deng, Ziqing Lu, Minghui Fu, Jianyu Su

**Affiliations:** 1 Bioengineering Department, Biological and Pharmaceutical College, Guangdong University of Technology, Guangzhou 510006, China; 2 School of Food Science and Engineering, South China University of Technology, Guangzhou 510640, China

**Keywords:** biosynthesis, *Cinnamomum burmannii*, D-borneol, third-generation sequencing and second-generation sequencing

## Abstract

*Cinnamomum burmannii* is a cinnamomum plant rich in natural D-borneol. Natural D-borneol is a bicycle monoterpenoid compound widely used in the food, pharmaceutical, and cosmetic industries. Therefore, analyzing the biosynthesis mechanism of natural D-borneol in *C. burmannii* at the molecular level is helpful for directional breeding in the future and further development and utilization of *C. burmannii* and its related gene resources. In our study, 76 genes related to terpene metabolism were analyzed through third-generation sequencing and second-generation sequencing. Of these genes, 57 were associated with the synthesis of the terpenoid skeleton, and 19 belonged to terpenoid synthase, including four monoterpenoid synthases, seven sesquiterpenoid synthases, and eight diterpenoid synthases. Two genes in diterpenoid synthase were differentially expressed in high D-borneol and low D-borneol plants. It was speculated that these two genes might be related to D-borneol synthesis. How these two genes participate in the synthesis of D-borneol needs further study.

## 1. Introduction

*Cinnamomum burmannii* belongs to the *Cinnamomim* genus of the Lauraceae family, which is distributed in Guangdong, Guangxi, Yunnan, and Fujian in China, India, and Indonesia [1]. Its branches and leaves are rich in terpenoids, such as D-boreol, eucalyptol, and pineol [2], etc. These terpenoids have essential uses in the food, pharmaceutical, and cosmetic industries. For example, D-boreol is a cyclic monoterpenoid with antibacterial, anti-tumor, anti-inflammatory, and analgesia properties, enhancing drug absorption and changing the blood–brain barrier effects [3,4,5]. However, different plants originated from other regions or lived in different seasons, with even different tissues in the same plant, and the type and content of terpenoids vary considerably [6]. This is related to the in vivo regulation of terpenoid biosynthesis. We must first resolve the molecular mechanism of terpenoid biosynthesis in *C. burmannii* to understand the regulation of terpenoid synthesis. The analysis of genes related to terpenoid biosynthesis in *C. burmannii* can help to reveal the molecular mechanism of terpenoid biosynthesis, provide theoretical guidance for directional breeding at the molecular level, and also lay a foundation for the further development and utilization of crucial gene resources of terpenoid biosynthesis in *C. burmannii* in the future.

According to previous studies on the biosynthesis and metabolism of terpenoids in plants [7,8,9], 5-carbon skeleton isopentenyl pyrophosphate (IPP) and dimethylallyl diphosphate (DMAPP) are prerequisites for the synthesis of terpenoids. Mevalonate (MVA) and 2-C-methyl-D-erythritol-4-phosphate (MEP) are the two major pathways for the biosynthesis of IPP and DMAPP in plants. In the MVA pathway, the biosynthesis of IPP in cytoplasm begins with two acetyl-CoA, through acetyl-CoA C-acetyltransferase (AAT), hydroxymethylglutaryl-CoA synthase (HMGS), hydroxymethylglutaryl-CoA reductase (HMGR), mevalonate kinase (MK), phosphomevalonate kinase (PMK), diphosphomevalonate decarboxylase (MVAPP), and other enzymes. In the MEP pathway, the biosynthesis of IPP in plastids begins with pyruvate and glyceraldehyde-3-phosphate, through 1-deoxy-D-xylulose-5-phosphate synthase (DXS), 1-deoxy-D-xylulose-5-phosphate reductoisomerase (DXR), 2-C-methyl-D-erythritol 4-phosphate cytidylyltransferase (CMS), 4-diphosphocytidyl-2-C-methyl-D-erythritol kinase (CMK), 2-C-methyl-D-erythritol-2,4-cyclodiphosphate synthase (MCS), (E)-4-hydroxy-3-methylbut-2-enyl-diphosphate synthase (HDS), 4-hydroxy-3-methylbut-2-en-1-yl diphosphate reductase (HMBPP) and other enzymes. Then, IPP is converted into DMAPP under the catalysis of isopentenyl-diphosphate delta-isomerase (IDI) and divalent metal ions. IPP and DMAPP are catalyzed by prenyltransferase into geranyl diphosphate (GPP), the precursor of monoterpenoid biosynthesis. GPP is catalyzed by farnesyl diphosphate synthase (FPS) into farnesyl diphosphate (FPP), the precursor of sesquiterpenoid and triterpenoid biosynthesis. FPP is catalyzed by geranylgeranyl diphosphate synthase (GGPPS) into geranylgeranyl diphosphate (GGPP), the precursor of diterpenoid biosynthesis. Therefore, studying these genes of key enzymes and their expression characteristics is the focus of this research in exploring the mechanism of terpenoid biosynthesis.

Transcriptome sequencing has increasingly been used to decipher the metabolic pathways and regulatory networks of organisms [10,11]. The most commonly used methods are third-generation sequencing (full-length transcriptome sequencing) and second-generation transcriptome sequencing (RNA-seq) [12]. Illumina is the primary platform for RNA-seq [13]. It transcribes RNA into small, short fragments for reverse transcription and sequencing and then splices them by mapping them to full-length transcripts with the bioinformatical method. The full-length transcripts can be obtained by PacBio, a third-generation sequencing technology, to reconstruct a transcriptome directly using single-molecule real-time sequencing technology (SMRT) without the reference genome sequence [14]. However, third-generation sequencing cannot obtain gene expression information. Therefore, combing third-generation and second-generation sequencing is commonly used in studying differential gene expression without a reference genome sequence.

Here, we sequenced the full-length transcriptome of *C. burmanni* leaves and compared the expression profiles of leaf tissues from two different chemotypes of *C. burmanni.* Through the comparative analysis of genes in the biosynthesis and metabolism of terpenoids in the Kyoto Encyclopedia of Genes and Genomes (KEGG), genes related to the biosynthesis of terpenoids in *C. burmanni* were obtained, and differentially expressed genes (DEGs) were further analyzed. Our study could lay the foundation for further cloning and functional research of critical genes for terpenoid biosynthesis and reveal the molecular mechanism of terpenoid biosynthesis in *C. burmanni*.

## 2. Materials and Methods

### 2.1. Plant Materials

Three biological replicate samples of leaf tissue from Mei Pian Tree (M1, M2, and M3, the high D-borneol chemotype of *C. burmannii*) were provided by Guangdong Huaqingyuan Limited Corporation. Three biological replicate samples of leaf tissue from Yin Xiang (Y1, Y2, and Y3, the low D-borneol chemotype of *C. burmannii*) were collected at Guangdong University of Technology in April 2020. The leaf tissue sample (one biological replicate) for the full-length transcriptome sequencing was the same as the first sample from the Mei Pian Tree.

### 2.2. Determination of Chemical Compositions

D-borneol was extracted from *C. burmannii* using the Soxhlet method, as in our previous report [15]. The solid–liquid ratio of the fresh leaf samples to anhydrous ethanol was 1:60 (g/mL), the extraction temperature was 80 °C, and the extraction time was 6 h. The essential oil was diluted to a constant volume of 100 mL for gas chromatography–mass spectrometry (GC-MS). Gas chromatography analysis was conducted using an Agilent 7890A Gas Chromatograph (chromatographic column: Agilent 19091N-113, 30m × 320 μm × 0.25 μm). Nitrogen was used as the carrier gas with a 2 mL/min flow rate. GC procedure: kept at 70 °C for 1 min, elevated to 100 °C at 3 °C/min, and then raised to 250 °C at 15 °C/min, maintained for 1 min. The injection volume was 1.0 µL and without a spill. The injection port temperature was 220 °C, and the detection temperature was 230 °C.

Gas chromatography–mass spectrometry was conducted using a Shimadzu QP2010 PLUS GC/MS instrument (chromatographic column: SH-RXI-5SILMS, 30 m × 0.25 mm × 0.25 μm). The GC column temperature program was the same as the above. The injection port temperature was 280 °C, the EI ion source was 200 °C, and the connection line was 250 °C. The MS scan range (m/z) was 29–500.

### 2.3. RNA Extraction, cDNA Library Construction, and Sequencing

Total RNA was extracted by grinding the tissue in TRIzol reagent (Life Technologies, Carlsbad, CA, USA) on dry ice and processed following the protocol provided by the manufacturer. RNA quality was assessed on an Agilent 2100 Bioanalyzer (Agilent Technologies, Palo Alto, CA, USA) and checked using RNase-free agarose gel electrophoresis. The purity and concentration of the RNA were determined with a Nanodrop micro-spectrophotometer (Thermo Fisher, Waltham, MA, USA). After total RNA was extracted, eukaryotic mRNA was enriched using Oligo (dT) beads, and rRNA was removed using a Ribo-ZeroTM Magnetic Kit (Epicentre, Madison, WI, USA). Total RNA samples with RIN ≥ 8.0 and 2.0 < OD260/280 < 2.2 were used for constructing the cDNA libraries in PacBio and Illumina sequencing.

### 2.4. PacBio SMRTbell Library Construction and SMRT Sequencing

The enriched mRNA was reverse-transcribed into cDNA using a Clontech SMARTer PCR cDNA Synthesis Kit. The number of PCR cycles was optimized for the downstream large-scale PCR reactions, which generated double-stranded cDNA. In addition, >4 kb size selection was performed using the BluePippinTM Size Selection System and mixed equally with the no-size-selection cDNA. Then, large-scale PCR was conducted for the next SMRTbell library construction. cDNAs were DNA damage repaired, end-repaired, and ligated to sequencing adapters. The SMRTbell template was annealed to a sequencing primer, bound to polymerase, and sequenced on the PacBio Sequel platform using P6-C4 chemistry with 10 h movies by Gene Denovo Biotechnology Co. (Guangzbou, China).

### 2.5. PacBio Sequence Data Processing

The raw sequencing reads of cDNA libraries were classified and clustered into transcript consensus using the SMRT Link v5.0.1 pipeline, supported by Pacific Biosciences. The parameters were set as “--min-length 50 --max-length 15,000-min-snr 2.5-min-rq 0.8”. Briefly, circular consensus sequence (CCS) reads were extracted from subread BAM files. Then CCS reads were classified into full-length non-chimeric (FL), non-full-length (nFL), chimeras, and short reads based on cDNA primers and the polyA tail signal. Short reads were discarded. Subsequently, the full-length non-chimeric (FLNC) reads were clustered by iterative clustering for error correction (ICE, a subprogram of SMRT Link v5.0.1 pipeline) software to generate the cluster consensus isoforms. Two strategies were employed to improve the accuracy of the PacBio reads. First, the non-full-length reads were used to polish the above-obtained cluster consensus isoforms using Quiver software to obtain the FL polished, high-quality consensus sequences (accuracy ≥ 99%). Second, the low-quality isoforms were further corrected using Illumina short reads obtained from the same samples using the LoRDEC tool (version 0.8, Pacific Biosciences, USA). Then, the final transcriptome isoform sequences were filtered by removing the redundant sequences with CD-HIT-v4.6.7 software using a threshold of 0.99 identities.

### 2.6. Illumina RNA-Sequencing Library Construction and Sequencing

The enriched mRNA was fragmented into short fragments using a fragmentation buffer and reverse-transcribed into cDNA with random primers. Second-strand cDNA was synthesized using DNA polymerase I, RNase H, dNTP, and a buffer. Then, the cDNA fragments were purified with a QiaQuick PCR extraction kit (Qiagen, Venlo, The Netherlands), end-repaired, poly (A) added, and ligated to Illumina sequencing adapters. The ligation products were size-selected by agarose gel electrophoresis, PCR amplified and sequenced using Illumina HiSeqTM 4000 by Gene Denovo Biotechnology Co. (Guangzhou, China).

### 2.7. Second-Generation Data Processing

Reads obtained from the sequencing machines included raw reads containing adapters or low-quality bases, which affected the following assembly and analysis. Thus, we removed reads containing adapters, more than 10% of unknown nucleotides (N), and more than 50% of low-quality (*q*-value ≤ 20) bases to obtain high-quality clean reads on FASTP (version 0.18.0). The high-quality clean reads were mapped to the full-length transcripts using RSEM [16] (version 1.2.19, USA), and the gene abundances were calculated and normalized to reads per kb per million reads (RPKM). The formula was shown as follows:

$$\mathrm{RPKM}=\frac{10^{6}C}{NL/10^{3}}$$

Given RPKM(A) to be the expression of gene A, *C* to be the number of reads that are uniquely aligned to gene A, *N* to be the total number of reads that are uniquely aligned to all genes, and *L* to be the number of bases on gene A. The RPKM method can eliminate the influence of different gene lengths and sequencing data amounts on the calculation of gene expression. Therefore, the calculated gene expression could be used to compare the gene expression differences among samples. The RPKM value was calculated for each transcription region to quantify its expression abundance and variations using RSEM software.

### 2.8. Differential Gene Expression Analysis

RNA differential expression analysis was performed by DESeq2 software between two different groups. Genes with false discovery rate (FDR) parameters below 0.05 and absolute fold change ≥2 were considered DEGs.

### 2.9. Functional Annotation of DEGs

To annotate the isoforms, isoforms were BLAST-analyzed against the NCBI non-redundant protein (Nr) database, the Swiss-Prot protein database, the Kyoto Encyclopedia of Genes and Genomes (KEGG) database, and the COG/KOG database with the BLASTx program at an E-value threshold of 1e−5 to evaluate sequence similarity with genes of other species. Gene ontology (GO) annotation was analyzed by Blast2GO software [17] with Nr annotation results of isoforms. Isoforms ranking the first 20 highest scores and no shorter than 33 high-scoring segment pair (HSP) hits were selected to conduct Blast2GO analysis. Then, the functional classification of isoforms was performed using WEGO software [18].

### 2.10. RT-qPCR Validation of DEGs

10 DEGs with higher expression were randomly selected for further quantitative reverse-transcription polymerase chain reaction (RT-qPCR) verification. The primers were designed using Primer Premier 5. The RT-qPCR was finished using the SYBR Green PCR Master Mix (Takara, Beijing, China) on the CFX96 Touch Deep Well platform (Bio-Rad, USA) with a total reaction volume of 25 μL, comprising 2 μL cDNA, 12.5 μL SYBR Green qPCR Master Mix, 1 μL 10 μM each primer, and 8.5 μL nuclease-free water. The RT-qPCR procedure was as follows: 95 °C for 5 s, 39 cycles of 95 °C for 5 s, annealing temperature for 30 s, and then melted at 65 °C to 95 °C, increment 0.5 °C. The expression levels of these genes were analyzed using the 2−ΔΔCt method [19]. The values were expressed as means ± standard error. The elongation factor 1 (EF1) (Isoform0005426) was used as the internal reference gene in order to normalize the PCRs for the amount of RNA added to the reverse transcription reactions. The expression level of one gene was calculated relative to the other gene which was used as the control. So, the data were presented as the fold change in gene expression normalized to the reference gene and relative to the control. The significance of the difference among two chemotypes was tested by performing an Independent T-test on SPSS 11.0 software (IBM, Armonk, NY, USA).

### 2.11. Phylogenetic Analysis of Terpenoid Synthases

The monoterpenoid synthase, diterpenoid synthase, and sesquiterpenoid synthase genes analyzed above were translated into amino acids and used to BlastP against the non-redundant protein sequence database of NCBI to select more similarity sequences (E < 1e−5). We built a phylogenetic tree of these sequences using Mega-X [20]. The tree construction method was Maximum Likelihood, and bootstrap was used to judge branch reliability. The number of bootstrap replications was set to 1000. Other parameters were set to default values.

## 3. Results

### 3.1. Determination of D-Borneol in C. Burmanni

**Figure 1:**
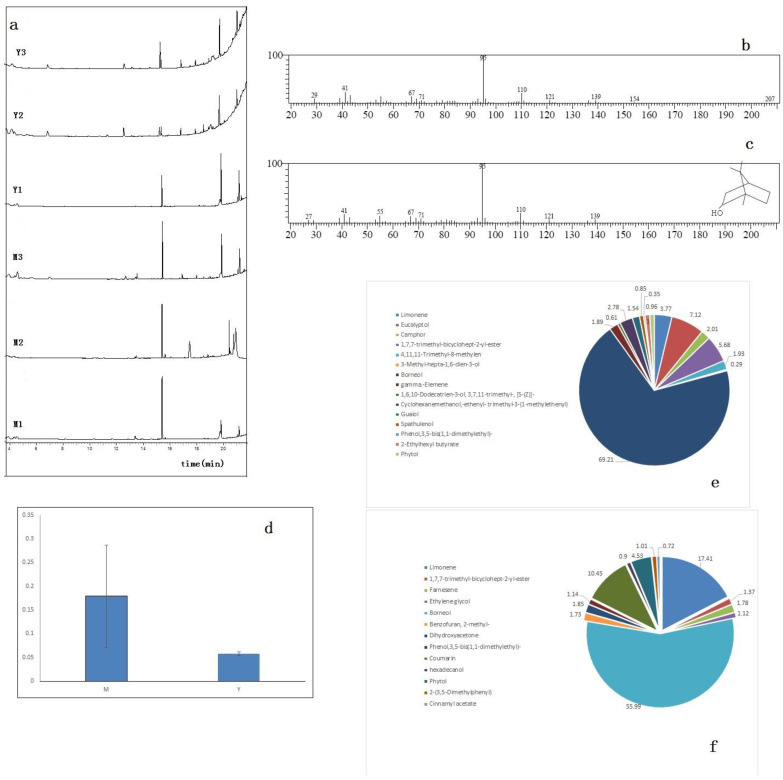
Determination of D-borneol content in two chemotypes of *C. burmannii* extraction. (**a**) Gas phase diagrams of extractions from the six samples. (**b**) Mass spectrum of D-borneol from *C. burmannii* samples. (**c**) Mass spectrum of D-borneol standard. (**d**) The contents of D-borneol in two chemotypes of *C. burmannii*, determined by GC method. (**e**) The percentage of extractions in the high D-borneol chemotype. (**f**) The percentage of extractions in the low D-borneol chemotype.

D-borneol was extracted from two chemotypes of *C. burmannis* (the high D-borneol chemotype M1, M2, M3, and the low D-borneol chemotype Y1, Y2, Y3) via Soxhlet apparatus, and the extraction was detected by GC-MS (Figure 1a–c). According to the peak area, the content of D-borneol in the high chemotype was about seven times that in the low chemotype (Figure 1d). A total of 15 compounds were detected in the high D-borneol chemotype, and the D-borneol content accounted for 66.21%. In the low D-borneol chemotype, 13 compounds were detected, and D-borneol accounted for 55.99% (Figure 1e,f).

### 3.2. RNA Sequencing and Transcriptomic Assembly

**Figure 2:**
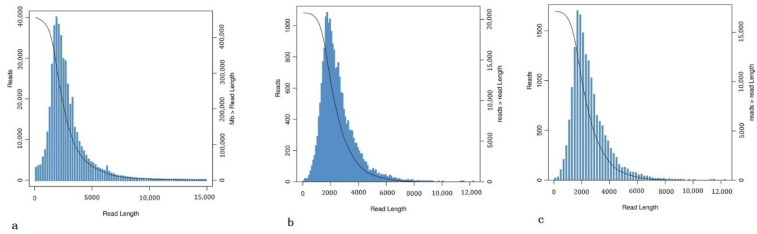
The length distribution map. (**a**) CCS length distribution. (**b**) Polished consensus isoforms length distribution. (**c**) The length distribution after redundancy removal.

The full-length transcriptome analysis of *C. burmanni* was conducted using single-molecule real-time (SMRT) sequencing on the PacBio Sequel platform. The offline data included 36,323,230,896 bp subreads, and the average length was 2181 bp. The N50 was 2610 bp. Sequences with the number of full passes more than or equal to 2 in the offline data were selected, and CCS in reads were extracted. In total, 458,065 reads of inserts (ROIs) were generated, with an average length of 2780 bp (Table 1; Figure 2a). Standard processing of the CCS sequences generated 232,454 full-length sequences, including 228,902 (98.47%) full-length non-chimeric reads (FLNC). After being polished by ICE and Quiver, the number of polished high-quality isoforms was 20,582, and the number of polished low-quality isoforms was 199 (Figure 2b). Then, CD-Hit-V4.6.7 was used to remove redundancy for subsequent analysis. After that, 17,116 isoforms were obtained. The average length was 2,506.84 bp, and the GC content was 44.04% (Table 2; Figure 2c). Quality control of raw reads was conducted with FASTP to filter low-quality data and clean the obtained reads. The relevant data for the six samples were summarized in Table 3. All data met the requirements and could be conducted in subsequent tests.

### 3.3. Gene Annotation and Functional Classification

**Figure 3:**
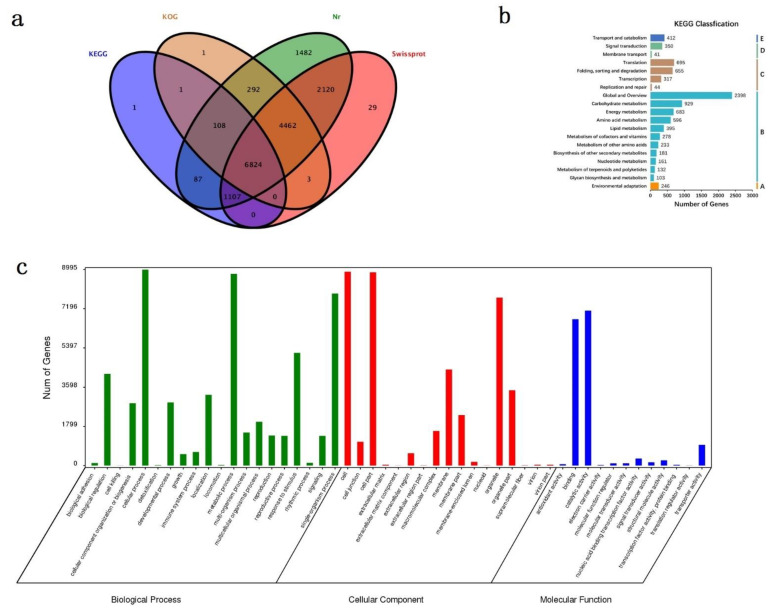
Gene annotation and functional classification. (**a**) Annotation information in 4 databases of the Venn diagram. (**b**) Annotation in KEGG pathway classification. The five KEGG metabolic pathways: A. Organismal Systems; B. Metabolism; C. Genetic Information Processing; D. Environmental Information Processing; E. Cellular Processes. (**c**) GO classification of all isoforms. The three main categories: biological process (BP), cellular component (CC), and molecular function (MF).

After processing the raw data, we obtained 17,116 isoforms. These isoform sequences were aligned to non-redundant protein sequences (NR) of NCBI, Swissprot, KEGG, and COG/KOG using BLASTX. Out of 17,116 isoforms, 16,516 isoforms were annotated, accounting for 96.49%. Among these isoforms, 16,481 were observed in NR, 14,544 in Swissprot, 11,690 in COG/KOG, and 8127 in KEGG. In these isoforms, 6824 were annotated by all four databases, accounting for 41.32% (Figure 3a). When we annotated these isoforms with KEGG, we found that 8127 were involved in 19 metabolic pathways (Figure 3b). These isoforms were divided into five categories according to the KEGG metabolic pathway: (A) organismal systems, (B) metabolism, (C) genetic information processing, (D) environmental information processing, and (E) cellular processes. One hundred thirty-two isoforms related to terpenoid and polyketide metabolism were discovered in all genes. After GO annotation of the obtained isoforms, 49 biological function annotations were obtained under three categories (biological process, cellular component, and molecular function). In the biological process, the cellular, metabolic, and single-organism processes were among the 20 terms that accounted for high proportions. In the cellular component, the cell, cell part, and organelle part were among the 17 terms that accounted for high proportions. In the molecular function, the binding and catalytic activities were among the 12 terms that accounted for high proportions (Figure 3c).

### 3.4. Analysis of DEGs between Different Groups

**Figure 4:**
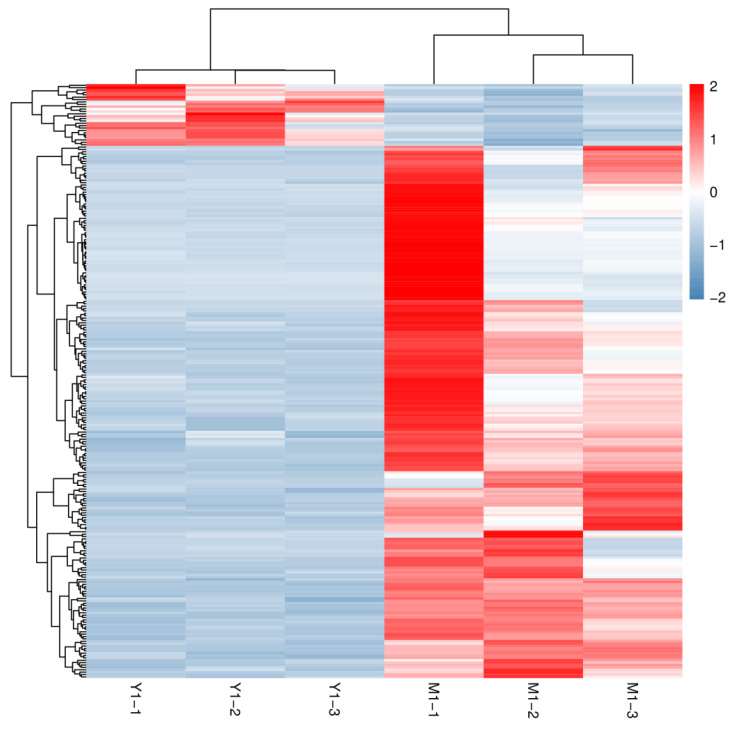
Comparison of differentially expressed genes in a heat map.

**Figure 5:**
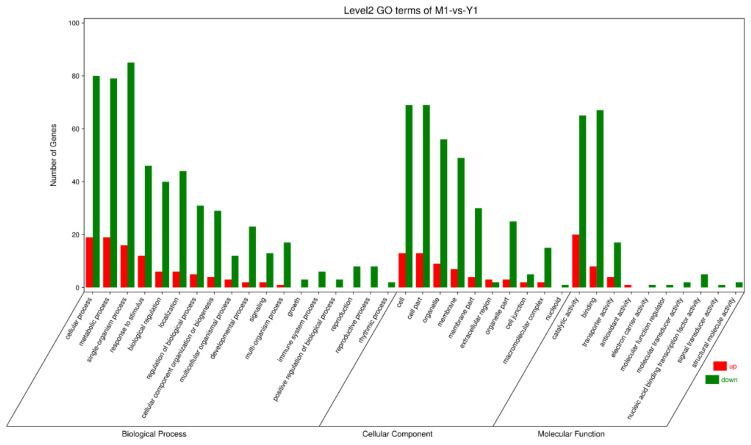
GO enrichment classification map of differentially expressed genes.

**Figure 6:**
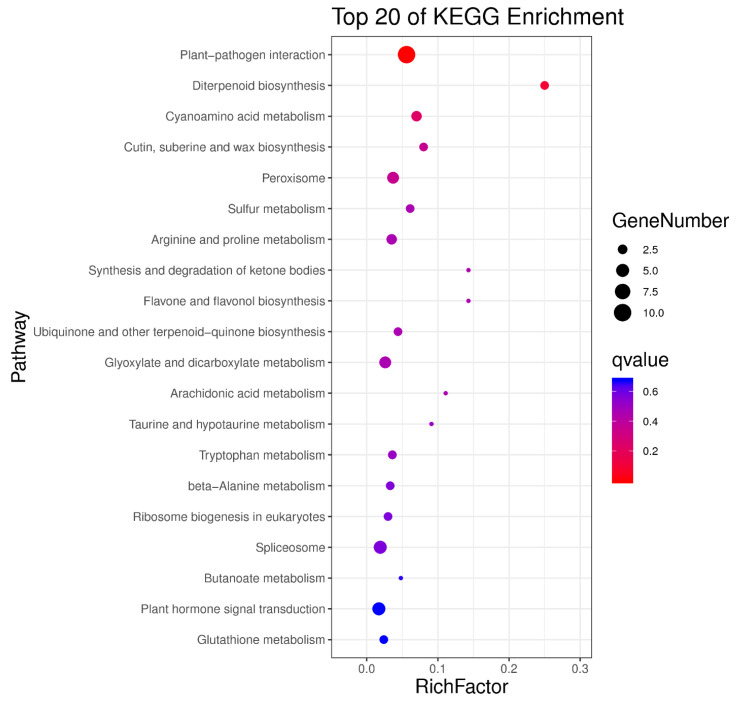
Bubble diagram of KEGG enrichment on differentially expressed genes.

The input data of gene differential expression analysis was the data of gene expression level obtained from the RNA-seq, which was analyzed using DESeq2, including the standardized probability of hypothesis testing. Such a probability was calculated according to the model to obtain the FDR value. Based on the results of the variance analysis, we screened FDR-value < 0.05 and |log_2_FC| > 1 (fold change) genes for DEGs. Comparing the M group with high D-borneol content, there were 26 up-regulated genes and 224 down-regulated genes in the Y group with low D-borneol content (the detailed FPKM values of differentially expressed genes were provided in Supplementary Table S1.M1-vs-Y1.filter.annotation). Differential gene expression patterns were hierarchically clustered, and a heat map (Figure 4) was used to present the clustering results. These genes with similar expression patterns may have common functions or may participate in common metabolic pathways and signaling pathways. The expression patterns of genes from the three Mei Pian Trees were similar, whereas those from the three Yin Xiangs were similar, but those of Mei Pian Trees and Yin Xiangs differed from each other (Figure 4). All of these DEGs were annotated by GO and KEGG analyses. According to the GO annotation analysis, the highest DEGs were involved in cellular, metabolic, and single-organism biological processes (Figure 5). In the cell components, the highest levels of DEGs were related to the components of the cell and cell parts. In the molecular function, the DEGs were concentrated on the binding and catalytic activities. According to the KEGG annotation (Figure 6), DEG in ko04626 had the most annotations. Such a DEG belongs to the interaction pathway between the plant and the pathogen in the biological system. It had annotated 10 DEGs, all of which had a downtrend. This annotation suggested that D-borneol biosynthesis might be related with the interaction of the plant and the pathogen.

### 3.5. Screening of Genes Related to Terpenoid Synthesis

**Figure 7:**
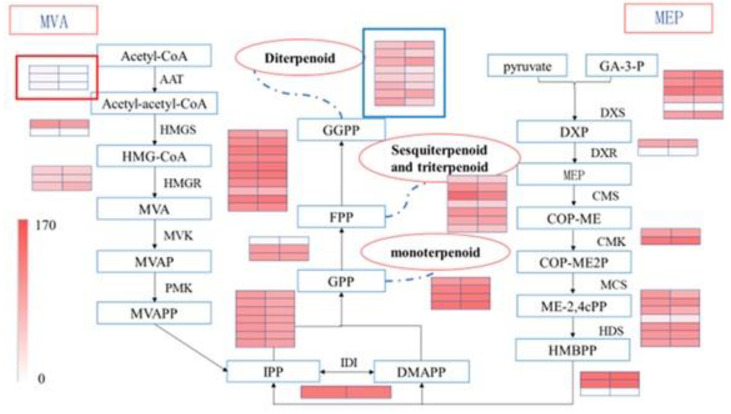
Expression of differentially expressed genes in the synthesis pathway of terpenoids in two chemical types of *C. burmannii*.

According to the existing studies, the biosynthesis pathways of terpenoids in plants are mainly acetyl-CoA via the MVA pathway and GA-3-P and pyruvate via the MEP pathway. In such pathways, IPP and different terpenoids are synthesized through a series of catalytic reactions. Based on the KEGG annotation, genes in the terpenoid synthesis pathways were further screened. As a result, 76 candidate isoforms encoded enzymes related to terpenoid biosynthesis. Among these 76 isoforms, 57 were related to carbon skeleton synthesis (Ko00900), 4 were related to monoterpenoid synthesis (Ko00902), 8 were related to diterpenoid biosynthesis (Ko00904), including 2 DEGs (isoform0014050 and isoform0016988), and the remaining 7 were related to sesquiterpenoid and triterpenoid biosyntheses (Ko00909). All these genes related to terpenoid biosynthesis were shown in Figure 7. Eight genes were annotated into the MVA pathway, including three AATs (a key enzyme in the MVA pathway), two HMGSs, and three HMGRs. In three AAT genes there was one DEG (isoform0010235). In the MEP pathway, six genes were associated with DXS, one of the most studied enzymes in the terpenoid biosynthesis pathway in plants. Two genes were related to the synthesis pathway of DXR, and the other two were annotated to CMK action. Seven sequences were associated with HDS. When HMBPP was converted into IPP, three related genes were used. Then, IPP used one associated gene during the isomer transformation with DMAPP. During GPP synthesis, seven related genes were annotated. Three annotated genes were used when the triterpenoid and sesquiterpenoid precursor FPP were synthesized. At the same time, 10 annotated genes were used during GGPP synthesis from FPP.

### 3.6. qRT-PCR Validation of DEGs from RNA-Seq Analysis

We randomly selected 12 isoforms with differentially expressed genes to verify the accuracy of the RNA-seq analysis. We optimized the annealing temperature of the primers and found that both the reference gene and the target genes had relatively appropriate Ct values, with a single dissolution peak at 56 °C (Table 4).

**Figure 8:**
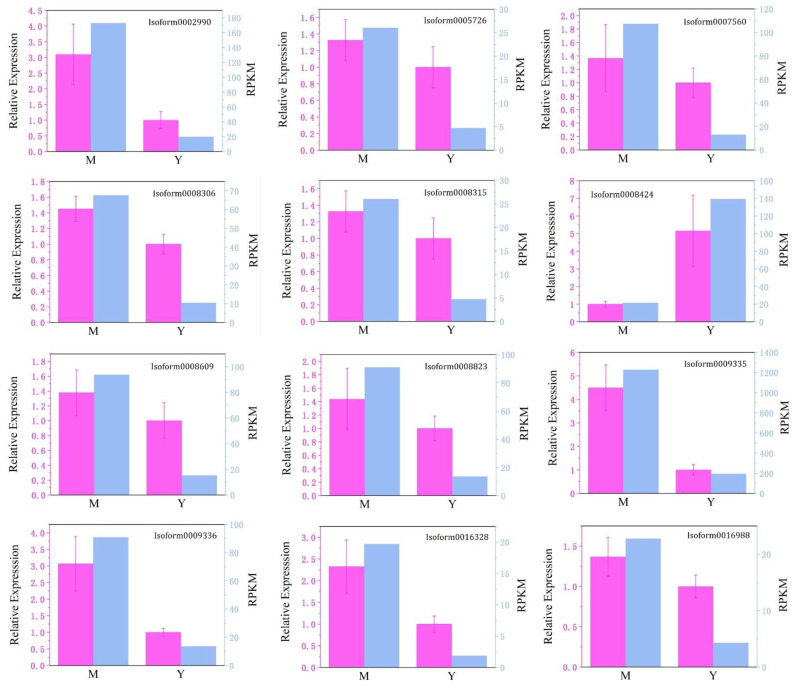
qRT-PCR validation of selected DEGs from the RNA-seq data. The relative expression levels shown in purple were estimated from the threshold of the PCR cycle with the delta delta CT method. The error bars indicate the standard error between 3 biological replicates. The FPKM data of the genes shown in blue bars were obtained by RNA-seq.

Standard curves were drawn using the reverse-transcribed cDNA at a 6-fold dilution at an annealing temperature of 56 °C, which were shown in the Supplementary Materials. According to the standard curves, the amplification efficiencies of the reference gene and all selected DEGs were between 90% and 110%. The determination coefficient (R^2^) was more significant than 0.980, and all chosen primers could be used in subsequent validation experiments. The location of the primers, the amplicon length, the predicted function and other information about the sequences were in the Supplementary Materials (highlighted in Table S1 and in Table S2). Amplification specificity was shown by a single peak in the melt peak curve. The melt peak curves and the amplification curves for all amplified genes were provided in the Supplementary Materials (File folder S2 and File folder S3). Although only four isoform (isoform0008424, isoform0009335, isoform0009336, isoform0016328) expressions were significantly different (Sig < 0.05) in Mei Pian Tree and Yin Xiang, the trend in the expression levels (Figure 8), showed that the RPKM value obtained by RNA-seq was basically consistent with that obtained by qRT-PCR, indicating the reliability of RNA-seq.

### 3.7. The Phylogenetic Analysis of Terpenoid Synthases

**Figure 9:**
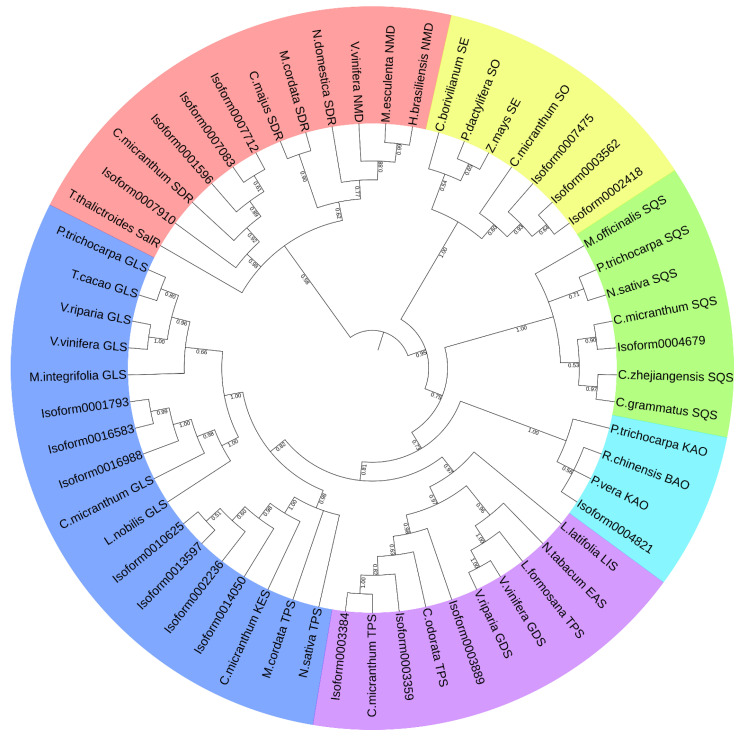
The result of phylogenetic analysis.

Similarity sequences from other plants involved in the phylogenetic analysis were listed in Table 5. The result of the phylogenetic analysis was shown in Figure 9. Four monoterpenoid synthase genes (Isoform0001596, Isoform0007083, Isoform0007712, and Isoform0007910) were clustered with short-chain dehydrogenase genes from *C. micranthum*. It was speculated that the function of these four genes may be related to dehydrogenation. Seven sesquiterpenoid synthase genes were clustered into three classes. This suggested that the functions of these seven sesquiterpenoid synthases were split into three categories. Among them, three (isoform0003899, isoform0003384, and isoform0003359) were clustered with sesquiterpenoid synthase genes from *C. micranthum*. One isoform (isoform00004679) was clustered with the squalene sesquiterpenoid synthase gene of *C. micranthum*. The other three (isoform0007425, isoform0003562, and isoform0002418) were associated with the squalene monooxygenase of *C. micranthum*. Eight diterpenoid synthase genes were also clustered into three categories. The isoform0014050, isoform0002236, isoform0013597, and isoform0010625 were classified with the ent-kaur-16-ene synthase of *C. micranthum*. Isorform0016988, isoform0016583, and isoform0001793 had clustered with diterpene geranyllinalool synthase, indicating that these two categories of diterpenoid synthase might have different functions from each other. The last diterpenoid synthase (isoform00004821) was clustered with beta-amyrin11-oxidase, indicating that this diterpenoid synthase differed from both above. Although the group of the last isoforms (isoform0003899, isoform0003384, and isoform0003359) was related to sesquiterpenoid synthase, it clustered with the diterpenoid synthase group.

## 4. Discussion

*Camphaceae* plants are rich in terpenoids, and different *Camphaceae* plants have different terpenoids [21], mainly due to their differences in vivo biosynthesis processes. The resolution to the biosynthesis of these terpenoids can lay a theoretical foundation for further effective development and utilization of these camphor plant resources. *C. burmannii* is rich in borneol, a double-loop monoterpenoid compound [1]. Borneol is a vital drug promoter in Chinese medicine, which can effectively promote drug absorption and has antibacterial and anti-tumor effects [3,4,5]. Therefore, the *C. burmannii* transcriptome analysis can provide evidence for studying the borneol biosynthesis.

Studies using transcriptome data to analyze plant secondary metabolites have been reported in some studies involving *Camphaceae*. Chen et al. [22] performed RNA sequencing to profile the leaf transcriptomes of linalool- and borneol-type chemotypes of *C. camphora*. Hou et al. [23] compared the transcriptome profiles of linalool-type *C. camphora* of high-oil-yield trees with low-oil-yield trees. They analyzed the essential regulatory genes involved in terpenoid biosynthesis in *C. camphora*. Jiang et al. [24] used RNA-seq to analyze the transcriptome of five chemotypes of *C. camphora* (linalool, camphor, cineol, iso-nerolidol, and borneol chemotype). Yan et al. [25] performed transcriptomic sequencing of *C. longepaniculatum* leaves to identify the factors involved in terpenoid metabolite biosynthesis. Yang et al. [26] mined the candidate genes involved in the biosynthesis of dextrorotatory borneol in *C. burmannii* using transcriptomic analysis of three chemotypes. However, these transcriptomic analyses were only based on the second-generation sequencing data, which were short and needed to be spliced into a unigene to predict the function. During splicing, missplicing and chimeras might arise due to the absence of reference genome sequences. The third-generation sequencing technology PacBio uses single-molecule real-time sequencing technology (SMRT) to obtain full-length transcripts without splicing due to its super-length read length (average 15 kb). Its false sequencing can be corrected with RNA-seq. The short sequences by RNA-seq could be mapped onto the sequences by PacBio. Therefore, high-quality transcripts can be obtained with a combination of third- and second-generation sequencing. It is also conducive to studying mRNA structures, such as variable shear, fusion genes, allele expression, etc. Thus, the study of full-length transcripts is becoming increasingly popular. Qiu et al. [27] analyzed the terpenoid synthases of *C. porrectum* based on a full-length transcriptome. They provided new clues for the functional exploration of the terpenoid synthesis mechanism and key genes in different chemotypes of *C. porrectum*.

Comparing the results of the second-generation sequencing alone and those of the second-generation sequencing combined with the third-generation sequencing, the former would obtain more sequences. Still, many were redundant, and some were fragments of the same gene, divided into multiple genes because they were not spliced. Therefore, the results of the second-generation sequencing alone were more sequences and shorter sequences, and most N50s were only a little longer than 1000 bp. Combined with the results of the third-generation sequencing, the sequences were long, and most N50s could reach more than 2000 bp [27]. For example, in Yang’s report [26], although 100,218 unigenes with an N50 of 1128 bp were collected in three different chemotypes of *C. burmannii*, only 45.38% of the unigenes were annotated. However, we obtained 17,116 isoforms with an N50 of 2749 bp in our study. Of these, 16,516 isoforms could be annotated, covering 96.5% of the total.

In this study, we used third-generation sequencing combined with second-generation sequencing to analyze the terpenoid synthase transcripts of *C. burmannii*. We found 76 genes related to terpenoid biosynthesis. Among them, 9 were involved in the MVA pathway genes, and 21 were involved in the MEP pathway. It was shown that the MEP pathway was the main pathway of terpenoid metabolism in *C. burmanni*. Although the MVA pathway may not be the main route of the carbon skeleton in the *Cinnamomum* plant, it may also play a key role in controlling synthetic precursor substances. Moreover, in our result, there was one DEG (isoform0010235) in MVA, which indicated that the MVA pathway in these two chemotypes was not same. Whether this difference may be related to the D-borneol biosynthesis and how MVA pathway regulates the biosynthesis of terpenoid precursors require further study. Our study obtained 19 terpenoid synthase genes, including 4 monoterpenoid synthases, 8 diterpenoid synthases, and 7 sesquiterpenoid synthases clustered into different groups in the phylogenetic analysis. Therefore, many terpenoid synthase genes indicated the existence of several terpenoid synthesis processes in *C. burmanni*, consistent with the component analysis results in the first part of our study. Among these terpenoid synthase genes, there were two DEGs (isorform0014050 and 0016988), indicating that the function of these two genes may be associated with borneol biosynthesis. Although sequence analyses of these two genes showed that they were diterpenoid synthase genes, whether they genuinely catalyze diterpene biosynthesis requires further experimental validation. The three kinds of terpenoid synthase (monoterpenoid synthase, diterpenoid synthase, and sesquiterpenoid synthase) did not differ significantly in the sequence. Small changes in the sequence at the active site in terpenoid synthase can lead to different products. Sometimes, the same enzymes can catalyze to obtain different products due to different substrates. Consequently, how these two differential diterpenoid synthases catalyzed the biosynthesis of borneol, a circular monoterpenoid, remains to be further studied.

From the phylogenetic analysis, we also found that the sesquiterpenoid synthase genes were clustered with the diterpenoid synthase genes, indicating that the clustering obtained with the sequences disagreed with the clustering obtained with the catalysate. This was consistent with previous reports about the phylogenetic analysis of the terpenoid synthase gene, in which the terpenoid synthase genes were clustered into five groups (TPS-a, TPS-b, TPS-c, TPS-e/f, and TPS-g) instead of being clustered into three groups (monoterpenoid synthase genes, diterpenoid synthase genes, and sesquiterpenoid synthase genes) [28,29]. This suggested that the catalytic products of terpenoid synthases were not only related to their encoding sequences, but might also be closely associated with substrates, reaction micro-environments, and other factors, which might also be reasons for terpene diversity in plants.

## Figures and Tables

**Table 1:** Summary of the SMRT sequencing data.

Item	Answer
Total base(bp)	36,323,230,896
average length	2181
N50	2610
Number of reads	458,065
Number of CCS bases	1,273,679,005
CCS Read Length (mean)	2780
Number of Passes (mean)	8
Number of unpolished consensus isoforms	20,796
2499.751	Mean unpolished consensus isoforms read length
Number of polished high-quality isoforms	20,582
Number of polished low-quality isoforms	199

**Table 2:** Isoform statistics after redundancy removal.

Total Number	Total Length (bp)	Maximum Length (bp)	Minimum Length (bp)	Average Length (bp)	N50 Length (bp)	GC Content (%)
17,116	42,907,024	12,292	86	2506.84	2749	44.04

**Table 3:** Short-sequencing statistics of different samples.

Sample	Raw Data (bp)	Clean Data (bp)	Q20	Q30	GC Content (%)
M1	7,228,626,300	7,187,544,129	6,927,661,015 (96.38%)	6,494,362,280 (90.36%)	48.56
M2	6,788,385,300	6,750,745,242	6,482,068,702 (96.02%)	6,041,734,157 (89.50%)	48.91
M3	7,040,023,800	6,999,531,017	6,741,621,103 (96.32%)	6,315,230,155 (90.22%)	48.85
Y1	6,238,760,400	6,201,129,378	5,953,219,753 (96.00%)	5,556,042,709 (89.60%)	48.46
Y2	6,938,253,600	6,898,066,477	6,616,909,592 (95.92%)	6,166,342,127 (89.39%)	48.47
Y3	6,646,860,900	6,620,075,722	6,377,116,047 (96.33%)	5,976,061,626 (90.27%)	49.17

**Table 4:** Genes for RT-qPCR and their primer sequences.

Gene Name	Primer Sequence	Amplification Efficiency (%)	R2	Annealing Temperature (°C)
Isoform0005426	F:AGACGGTTGCTGTTGGAGTT R: CACCATCCACCCCTTTGTCA	98.6	0.999	56
Isoform0002990	F: GGCATCAAGCCATCAACT R:AGAATCGCTGGAGAATCAT	101.7	0.997	56
Isoform0005726	F: GCATCCAAACTGGCAAGA R: CAATCGGAGAACCTCAAATA	95.4	0.999	56
Isoform0007560	F: GAAAGCAAGGGAAGAGGT R: GTGGATACAATCGGAGAAC	98.8	0.995	56
Isoform0008306	F:TAGTTCAGACGGCGAAGGAC R: GCTCAGATGCGGCAAGTG	95.5	0.997	56
Isoform0008315	F: GTATTCCCGTTGTTGATG R: ATTTGACCTAACCGTGCC	97.6	0.996	56
Isoform0008424	F: CGCCCTTCATCTCAACCT R: GCTCGTCCCACCAATACTT	97.5	0.992	56
Isoform0008609	F: GAAAGCAAGGGAAGAGGT R: GTGGATACAATCGGAGAAC	96.8	0.993	56
Isoform0008823	F: GAAAGCAAGGGAAGAGGT R: GTGGATACAATCGGAGAAC	100.6	0.999	56
Isoform0009335	F:TCAATAATCAGGGCAACACT R:TCTCATCCTACGCAACACC	99.8	0.996	56
Isoform0009336	F: ACTCCACCTTACTTTCATCT R: CTTATTAGCACGGTTTCC	99.9	0.998	56
Isoform0016328	F:GAGCGATGACAGTGAAAGCG R: CCTTCATCGGCCAAATCCCT	100.8	0.992	56
Isoform0016988	F: TCCACCCTTTTATCCCATC R: TGCTCTGACAAGCCCAAT	103.2	0.999	56

**Table 5:** Sequences involved in phylogenetic analysis other than *C. burmannii*.

Species Name	Accession No.	Gene Name	Rename
*Cinnamomum micranthum*	RWR72162.1	Short-chain dehydrogenase/reductase SDR	C.micranthum_SDR
*Thalictrum thalictroides*	KAF5191451.1	Salutarine reductase	T.thalictroides_SalR
*Chelidonium majus*	ACN87274.1	Short-chain dehydrogenase/reductase, partial	C.majus_SDR
*Morella rubra*	KAB1199009.1	Salutarine reductase	M.rubra_SalR
*Lavandula latifolia*	ABD77417.1	linalool synthase	L.latifolia_LIS
*Nandina domestica*	ACN87275.1	Short-chain dehydrogenase/reductase	N.domestica_SDR
*Hevea brasiliensis*	XP_021653042.1	(+)-neomenthol dehydrogenase-like	H.brasiliensis_NMD
*Vitis vinifera*	RVW27891.1	(+)-neomenthol dehydrogenase	V.vinifera_NMD
*Manihot esculenta*	XP_021615637.1	(+)-neomenthol dehydrogenase	M.esculenta_NMD
*Nicotiana tabacum*	AAA19216.1	5-epi-aristolochene synthase	N.tabacum_EAS
*Cinnamomum micranthum*	RWR82502.1	squalene oxygenase-like protein isoform X2	C.micranthum_SO
*Cinnamomum micranthum*	RWR82644.1	squalene synthase	C.micranthum_SQS
*Cinnamomum micranthum*	RWR94586.1	terpenoid synthase 1	C.micranthum_TPS
*Chlorophytum borivilianum*	AFN61200.1	squalene epoxidase	C.borivilianum_SE
*Zea mays*	ONL95392.1	squalene epoxidase 1	Z.mays_SE
*Phoenix dactylifera*	XP_038983584.1	squalene oxygenase SE1	P.dactylifera_SO
*Chimonanthus grammatus*	AYP73106.1	squalene synthase	C.grammatus_SQS
*Chimonanthus zhejiangensis*	AYP73108.1	squalene synthase	C.zhejiangensis_SQS
*Magnolia officinalis*	AMK48128.1	squalene synthase	M.officinalis_SQS
*Nigella sativa*	AMA66327.1	squalane synthase 1	N.sativa_SQS
*Populus trichocarpa*	XP_002313765.1	squalene synthase	P.trichocarpa_SQS
*Cananga odorata*	QMW48843.1	terpenoid synthase 2	C.odorata_TPS
*Liquidambar formosana*	AIO10964.1	TPS01	L.formosana_TPS
*Vitis riparia*	XP_034678035.1	(−)-germacrene D synthase-like	V.riparia_GDS
*Vitis vinifera*	RVW94686.1	(−)-germacrene D synthase	V.vinifera_GDS
*Cinnamomum micranthum*	RWR88021.1	terpene geranyllinalool synthase	C.micranthum_GLS
*Laurus nobilis*	AKQ19359.1	terpene geranyllinalool synthase	L.nobilis_GLS
*Vitis riparia*	XP_034698703.1	(E,E)-geranyllinalool synthase-like	V.riparia_GLS
*Macadamia integrifolia*	XP_042496091.1	(E,E)-geranyllinalool synthase	M.integrifolia_GLS
*Vitis vinifera*	NP_001268201.1	(E,E)-geranyllinalool synthase-like	V.vinifera_GLS
*Populus trichocarpa*	XP_024454968.1	(E,E)-geranyllinalool synthase	P.trichocarpa_GLS
*Theobroma cacao*	EOY28337.1	P(E)-nerolidol/(E,E)-geranyl linalool synthase	T.cacao_GLS
*Pistacia vera*	XP_031276291.1	ent-kaurenoic acid oxidase 1-like	P.vera_KAO
*Populus trichocarpa*	XP_002318613.3	ent-kaurenoic acid oxidase 1 isoform X3	P.trichocarpa_KAO
*Rosa chinensis*	XP_024174916.2	beta-amyrin 11-oxidase	R.chinensis_BAO
*Cinnamomum micranthum*	RWR88499.1	ent-kaur-16-ene synthase, chloroplastic isoform X1	C.micranthum_KES
*Macleaya cordata*	OVA15215.1	Terpenoid synthase	M.cordata_TPS
*Nigella sativa*	QLI42521.1	terpenoid synthase-like 2 protein, partial	N.sativa_TPS

## Data Availability

The transcriptome data of *C.* *burmannii* were deposited in GenBank of NCBI and assigned the accession numbers PRJNA810581 and PRJNA812411. All other data generated or analyzed during this study are included in this published article.

## Supplementary Materials

The following supporting information can be downloaded at: https://www.mdpi.com/article/10.3390/cimb44090288/s1, Table S1: M1-vs-Y1.filter.annot; Table S1: Sequences and their primers used in the RT-qPCR; File folder S1: Amplication curves; File folder S2: Melt peak curves; File folder S3: Standard curves.
