# Supplementary material for: Full-Length Transcriptome Sequencing Combined with RNA-Seq to Analyze Genes Related to Terpenoid Biosynthesis in Cinnamomum burmannii
Source: Curr Issues Mol Biol. 2022 Sep 12;44(9):4197–215. doi: 10.3390/cimb44090288 (PMC9497596; doi:10.3390/cimb44090288)
Supplement: Supplementary file 1 [file cimb-44-00288-s001.zip › new-supplementary materials/Table S2.pdf]

Table S2. Sequences and their primers used in the RT-qPCR.

Isoform	Length (bp)	Amplicon length (bp)	F primer location	R primer location	Gene Symbol	Gene Description
Isoform0002990	1855	119	1475-1492	1575-1593	CYP72A15	cytochrome P450 [Coptis japonica var. dissecta] [Coptis japonica]
Isoform0005726	1607	127	921-938	1028-1047	CYP72A15	cytochrome P450 [Coptis japonica var. dissecta] [Coptis japonica]
Isoform0007560	1330	116	662-679	759-777	CYP72A15	PREDICTED: cytochrome P450 CYP72A219-like [Nelumbo nucifera]
Isoform0008306	1148	122	693-712	797-814	DAD2	PREDICTED: probable strigolactone esterase DAD2 [Ricinus communis], sesquiterpenoid biosynthetic process
Isoform0008315	1164	142	412-429	536-553	Os01g0360600	PREDICTED: dephospho-CoA kinase [Amborella trichopoda]
Isoform0008424	1118	132	663-680	776-794	XTH9	PREDICTED: xyloglucan endotransglucosylase/hydrolase protein 9 [Nelumbo nucifera]
Isoform0008609	1064	116	396-413	493-511	CYP72A15	cytochrome P450 [Coptis japonica var. dissecta] [Coptis japonica]
Isoform0008823	954	116	286-303	383-401	CYP72A63	cytochrome P450 [Coptis japonica var. dissecta] [Coptis japonica]
Isoform0009335	444	137	162-181	280-298	--	-
Isoform0009336	457	191	48-67	221-238	--	-
Isoform0016328	2356	119	895-914	994-1013	DUF789	domain-containing protein [Cephalotus follicularis]
Isoform0016988	2266	81	171-189	234-251	diterpene	Metabolism

	geranyllinalool synthase [Laurus nobilis]
