# Supplementary figures and images for: Full-Length Transcriptome Sequencing Combined with RNA-Seq to Analyze Genes Related to Terpenoid Biosynthesis in Cinnamomum burmannii
Source: Curr Issues Mol Biol. 2022 Sep 12;44(9):4197–215. doi: 10.3390/cimb44090288 (PMC9497596; doi:10.3390/cimb44090288)

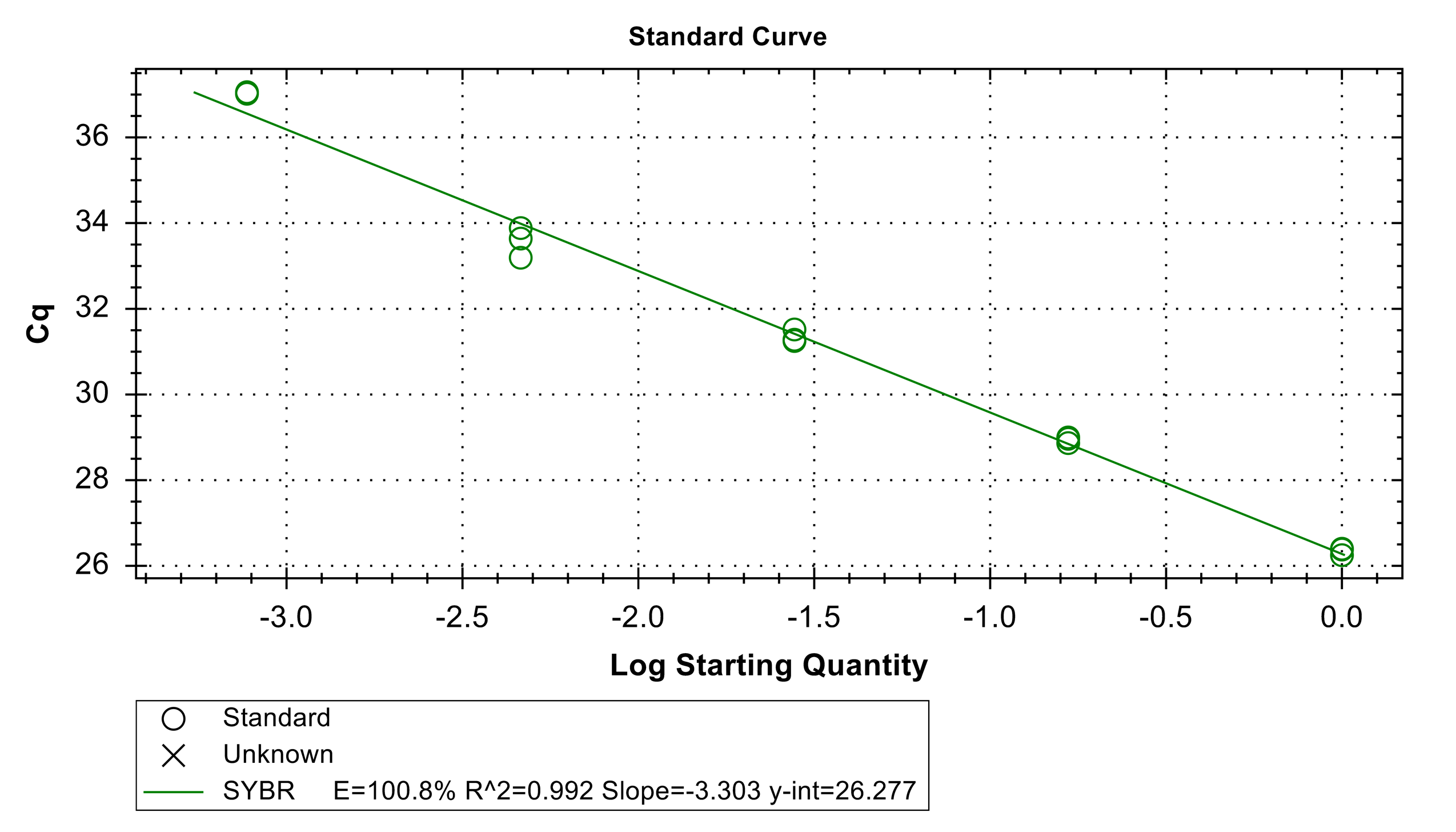

Supplement: Supplementary file 1 [file cimb-44-00288-s001.zip › new-supplementary materials/File folder S1.Standard curves/16328-56.png]

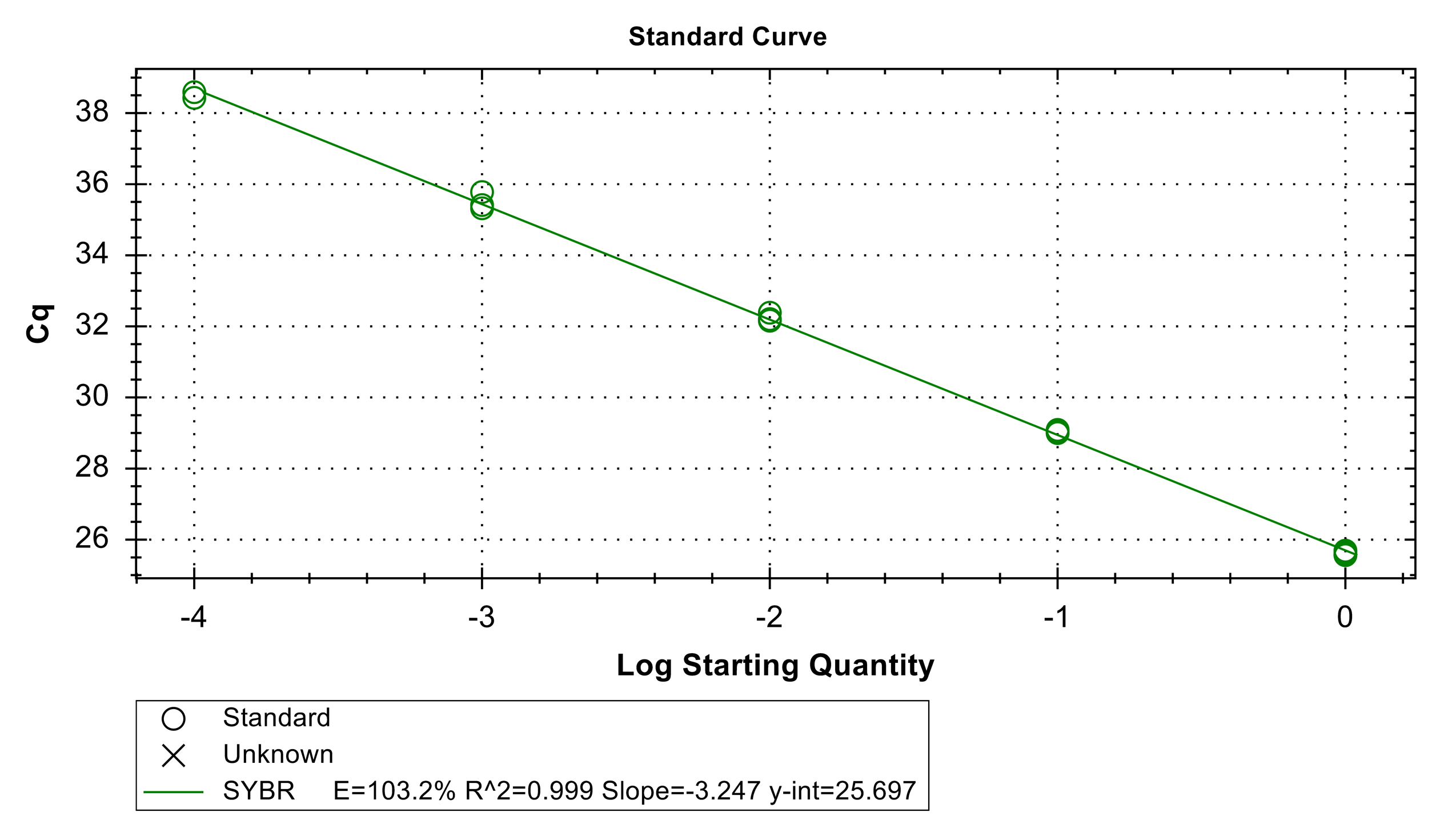

Supplement: Supplementary file 1 [file cimb-44-00288-s001.zip › new-supplementary materials/File folder S1.Standard curves/16988-56.png]

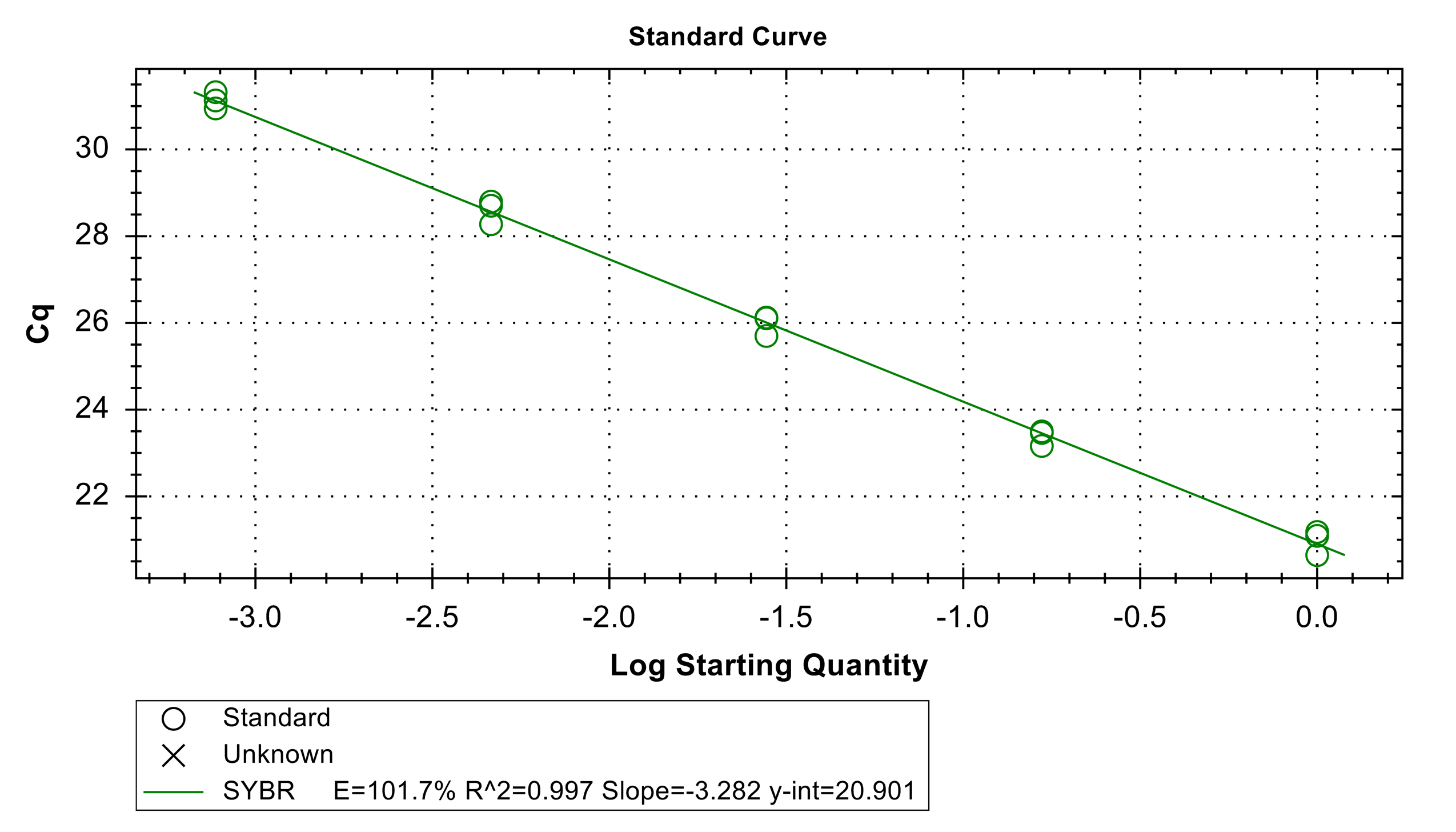

Supplement: Supplementary file 1 [file cimb-44-00288-s001.zip › new-supplementary materials/File folder S1.Standard curves/2990-56.png]

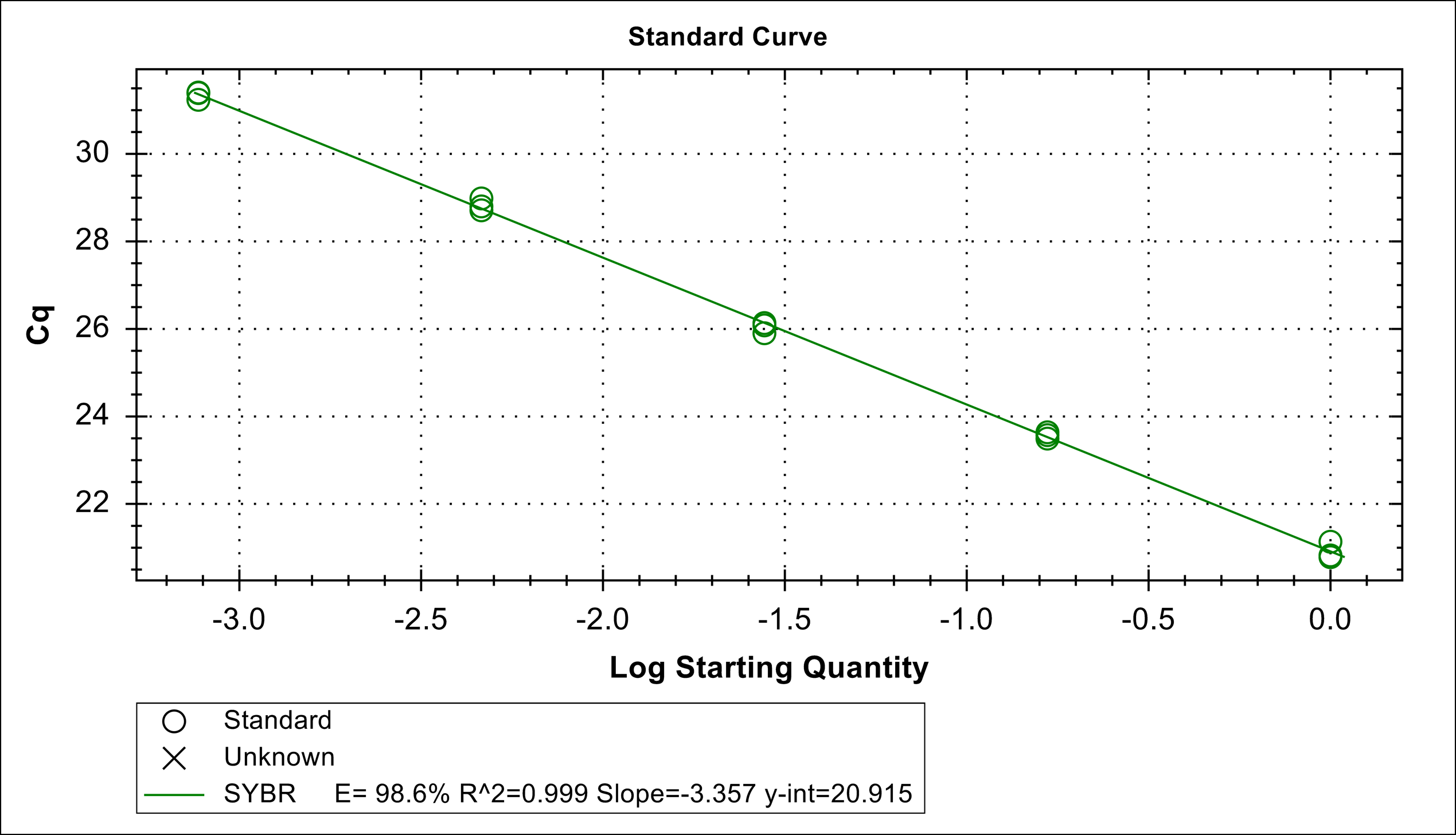

Supplement: Supplementary file 1 [file cimb-44-00288-s001.zip › new-supplementary materials/File folder S1.Standard curves/5426-56.png]

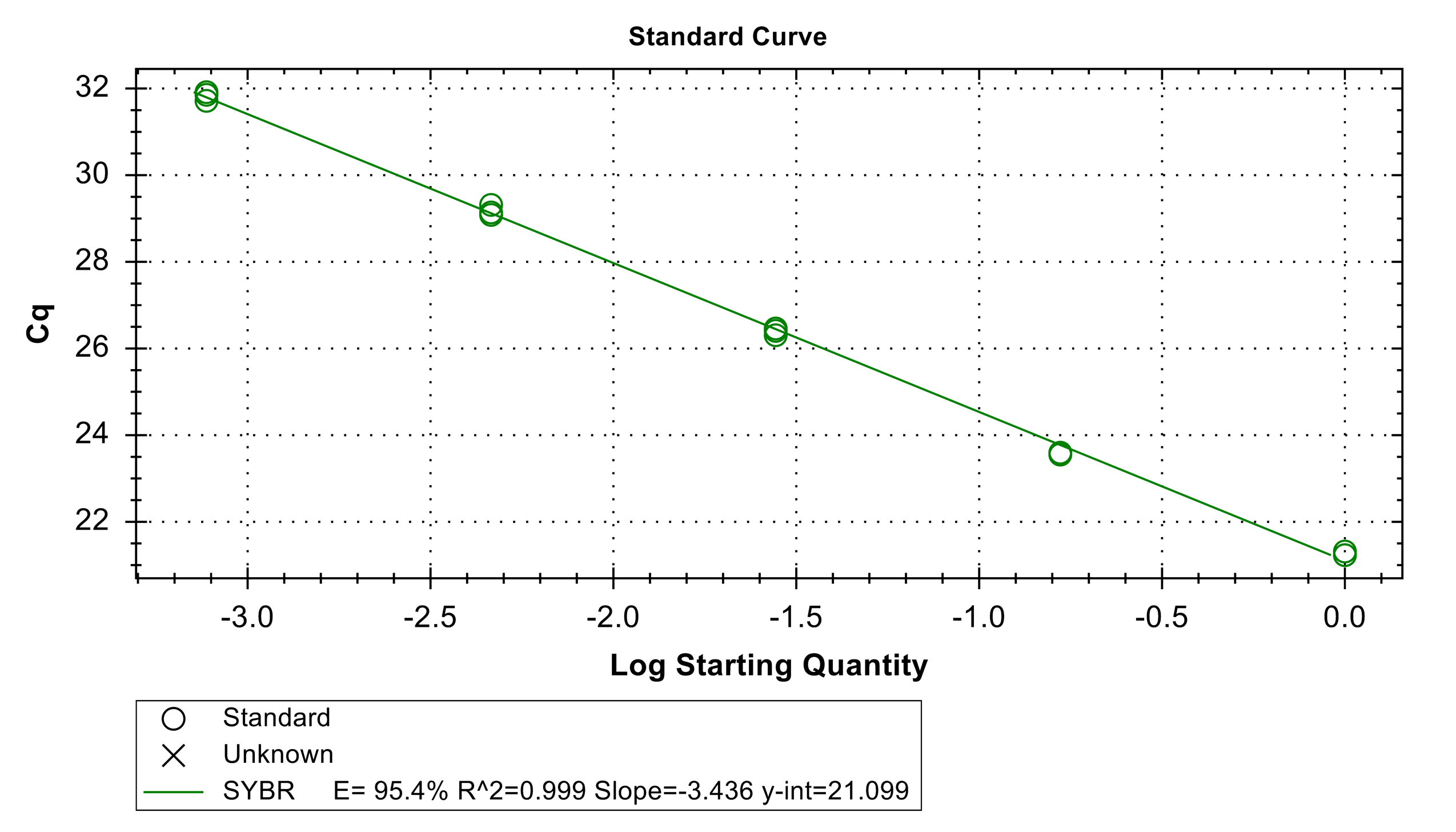

Supplement: Supplementary file 1 [file cimb-44-00288-s001.zip › new-supplementary materials/File folder S1.Standard curves/5726-56.png]

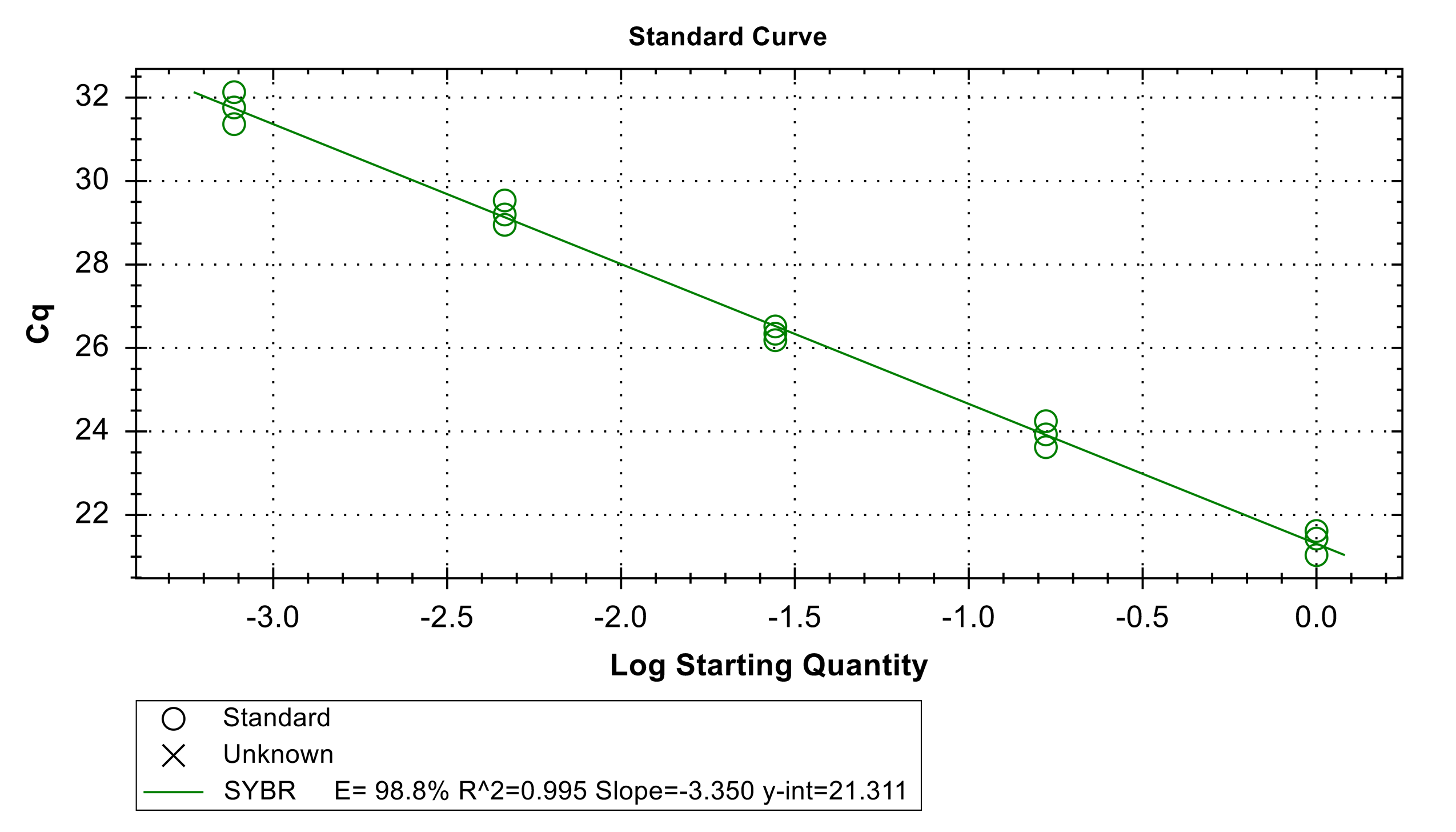

Supplement: Supplementary file 1 [file cimb-44-00288-s001.zip › new-supplementary materials/File folder S1.Standard curves/7560-56.png]

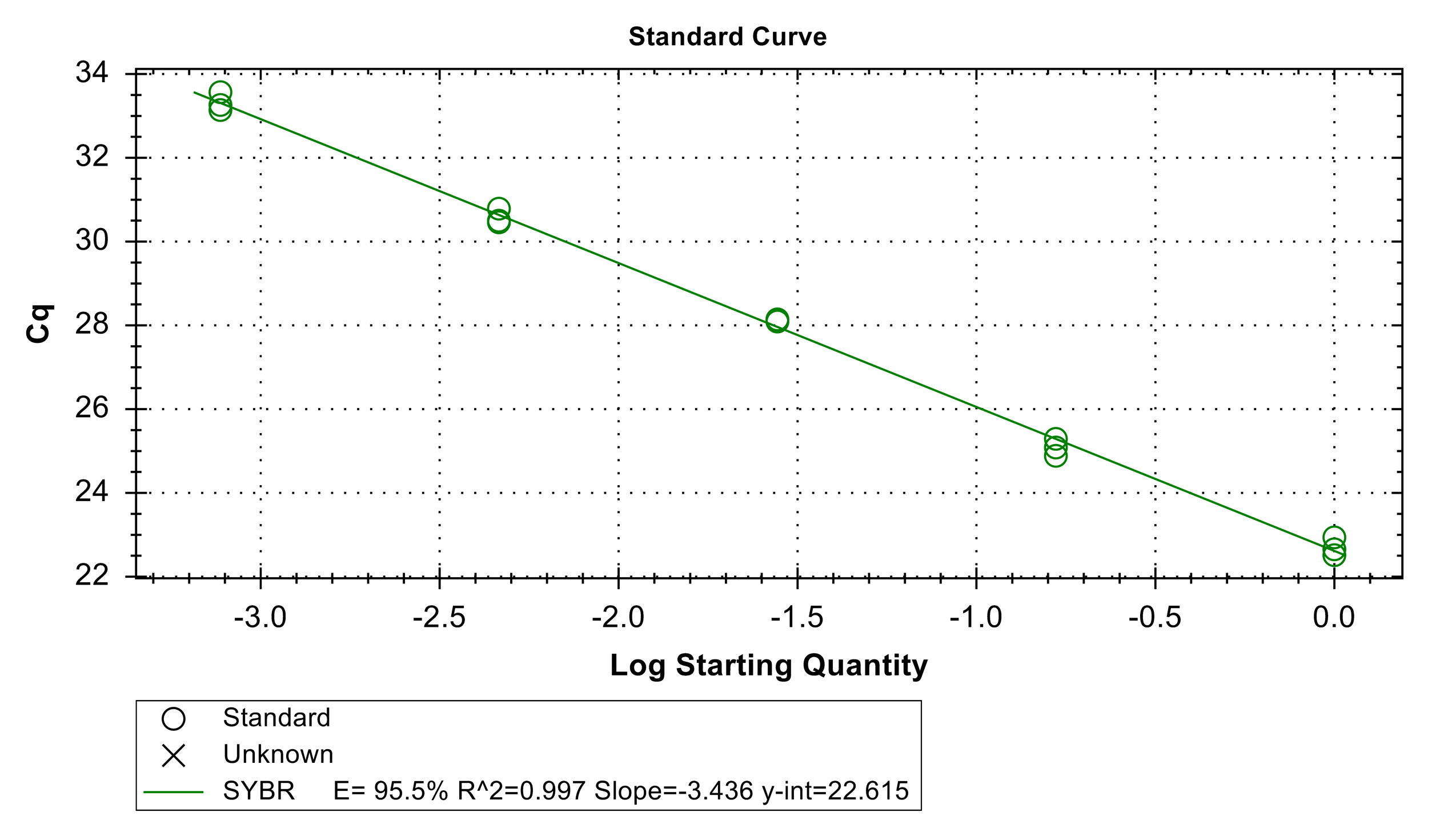

Supplement: Supplementary file 1 [file cimb-44-00288-s001.zip › new-supplementary materials/File folder S1.Standard curves/8306-56.png]

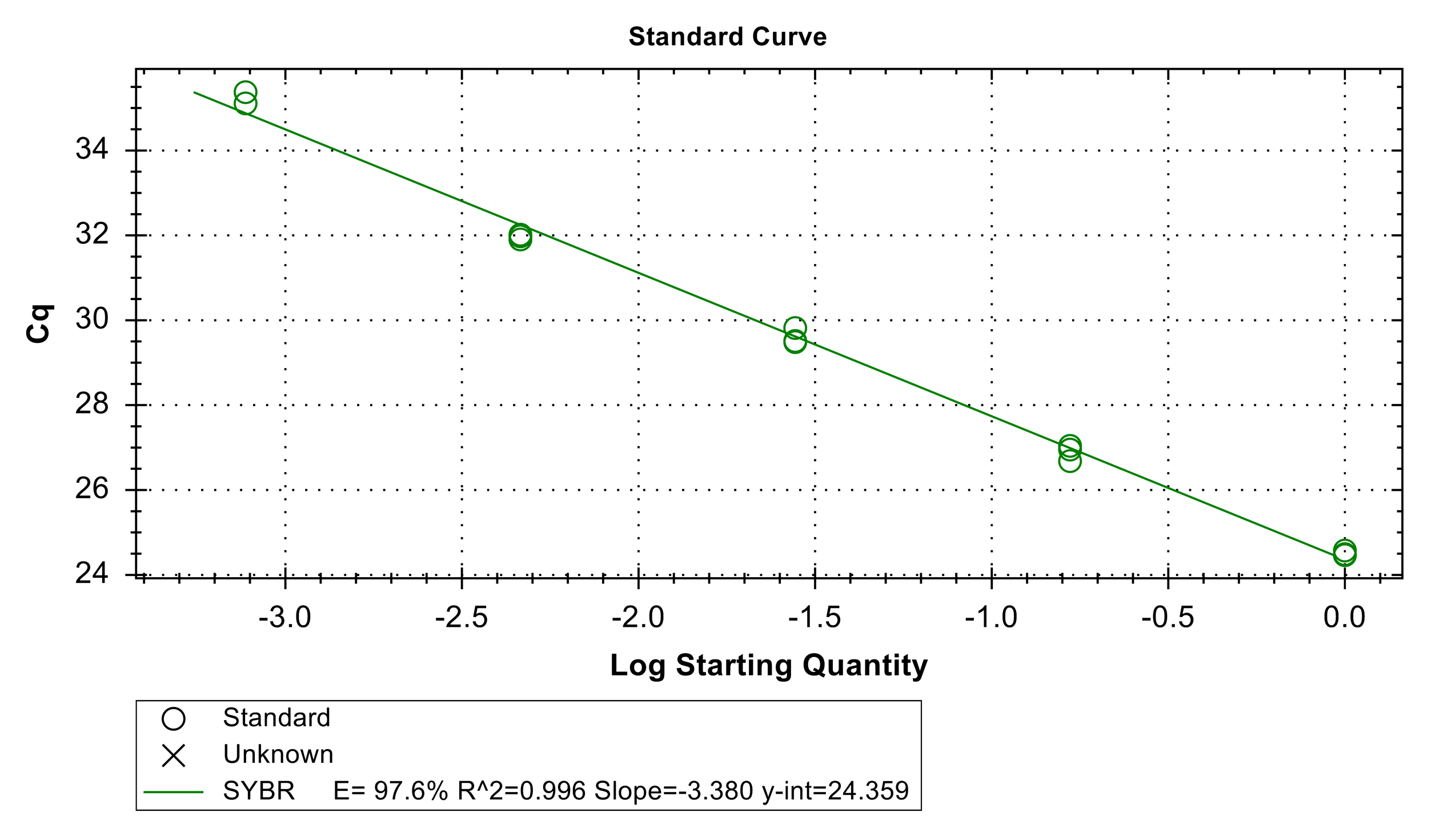

Supplement: Supplementary file 1 [file cimb-44-00288-s001.zip › new-supplementary materials/File folder S1.Standard curves/8315-56.png]

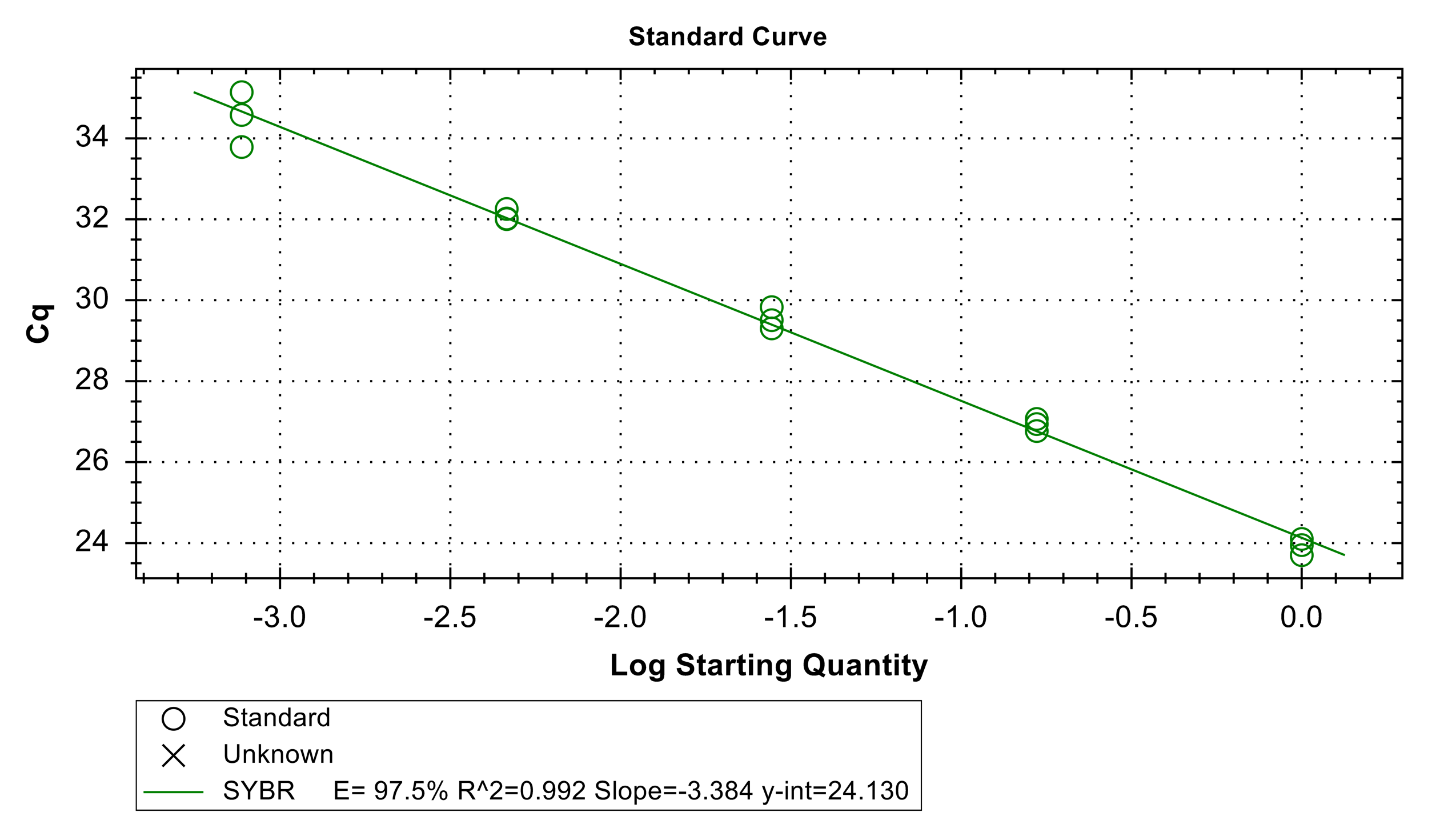

Supplement: Supplementary file 1 [file cimb-44-00288-s001.zip › new-supplementary materials/File folder S1.Standard curves/8424-56.png]

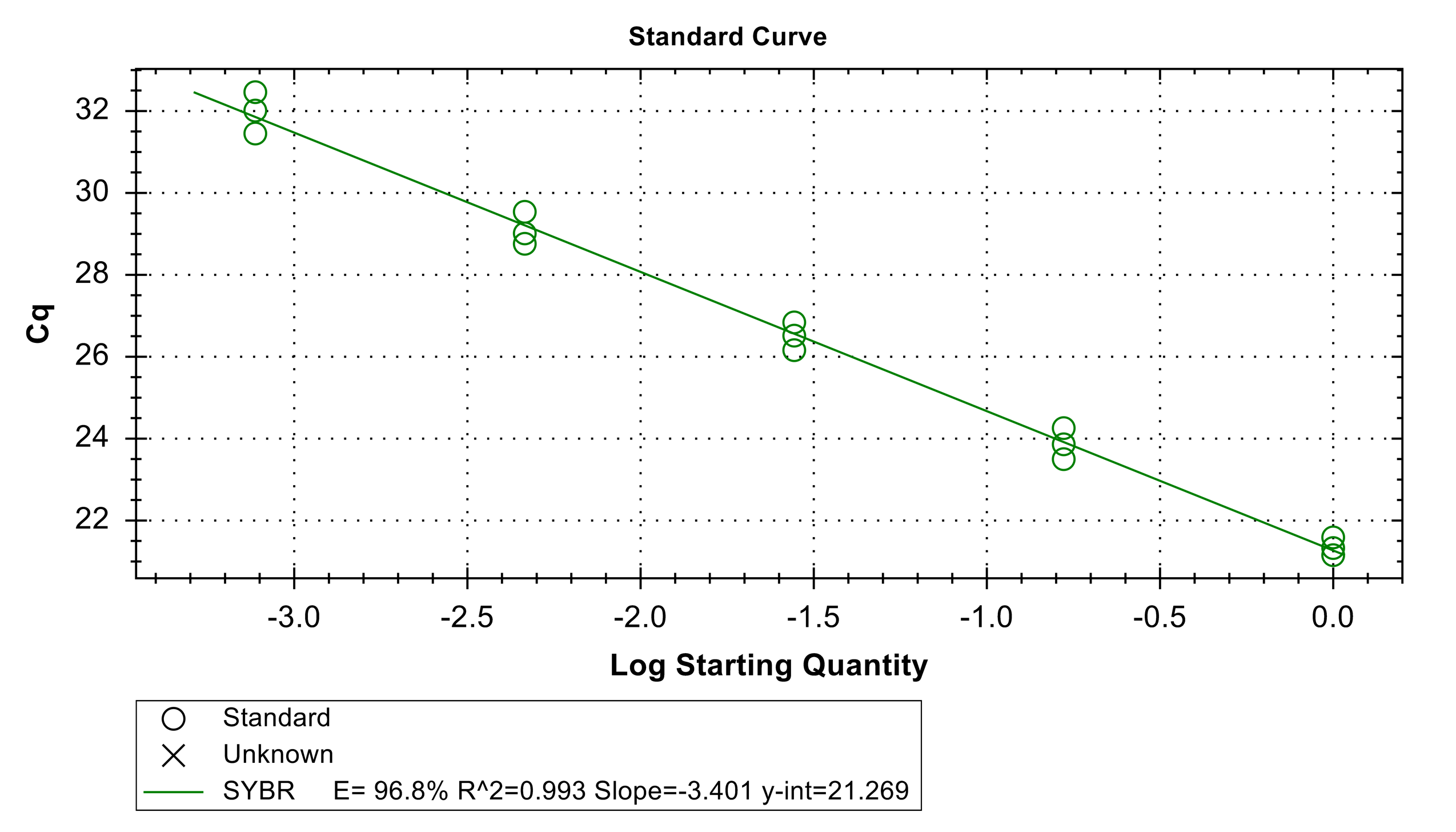

Supplement: Supplementary file 1 [file cimb-44-00288-s001.zip › new-supplementary materials/File folder S1.Standard curves/8609-56.png]

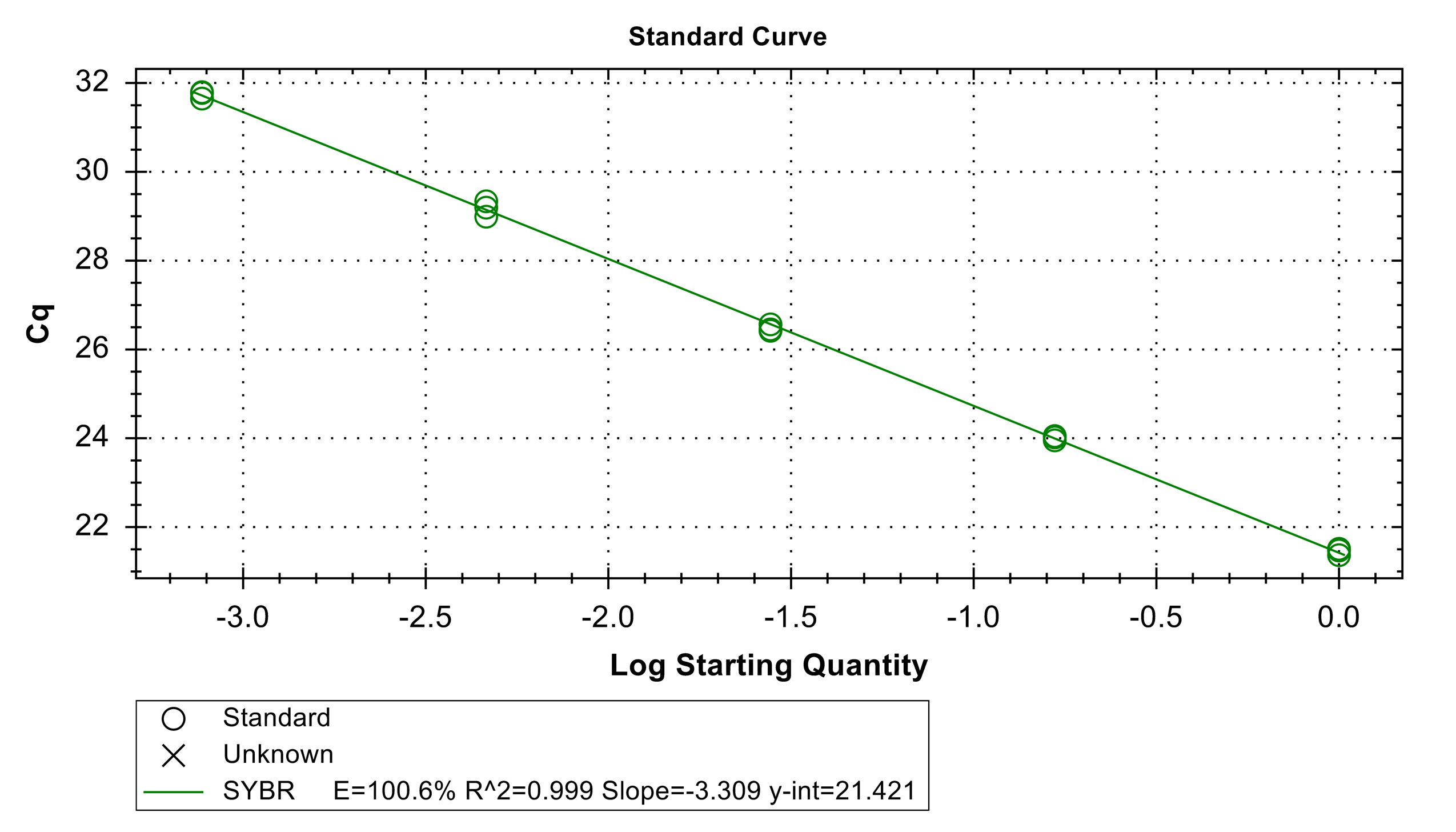

Supplement: Supplementary file 1 [file cimb-44-00288-s001.zip › new-supplementary materials/File folder S1.Standard curves/8823-56.png]

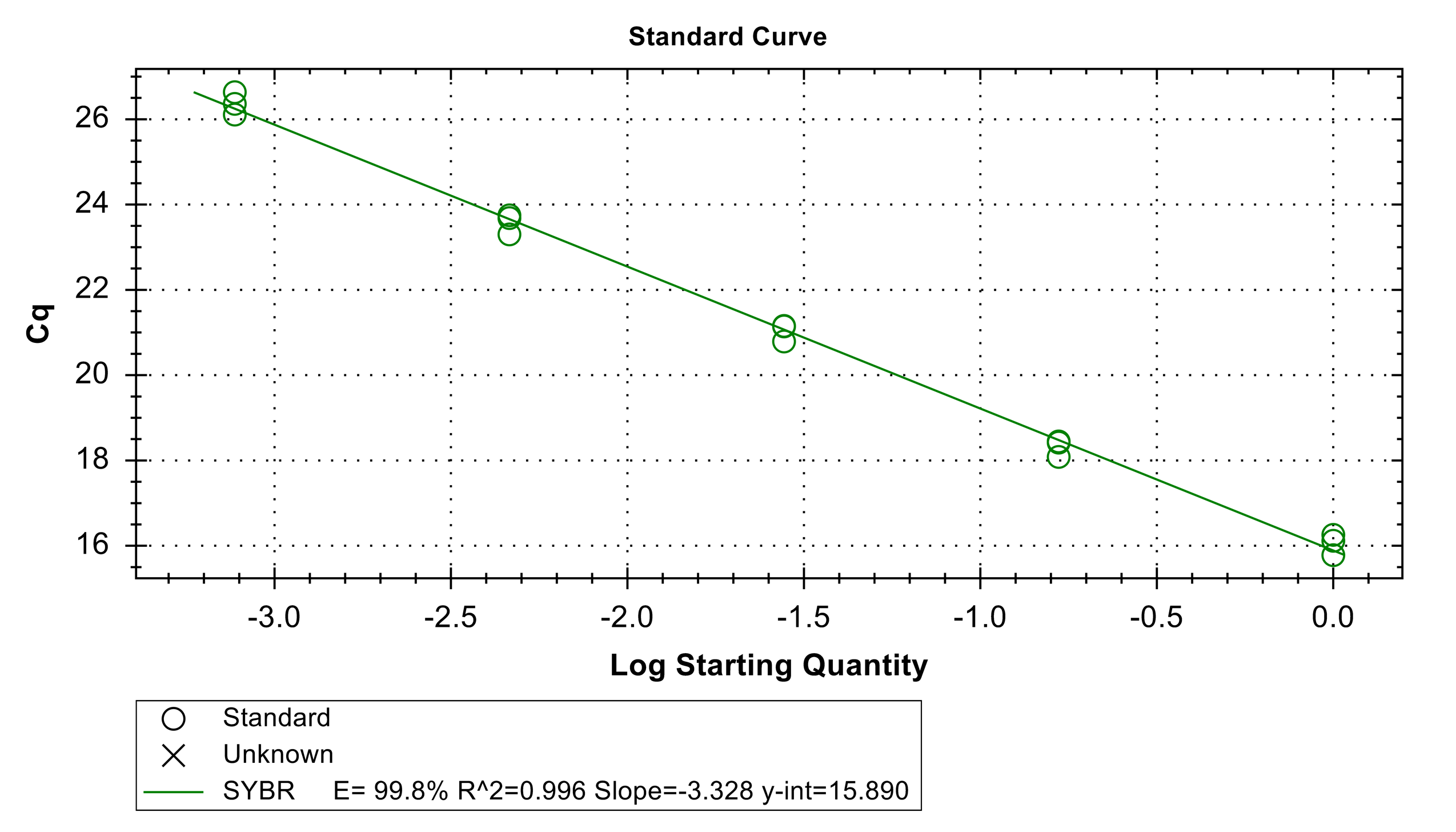

Supplement: Supplementary file 1 [file cimb-44-00288-s001.zip › new-supplementary materials/File folder S1.Standard curves/9335-56.png]

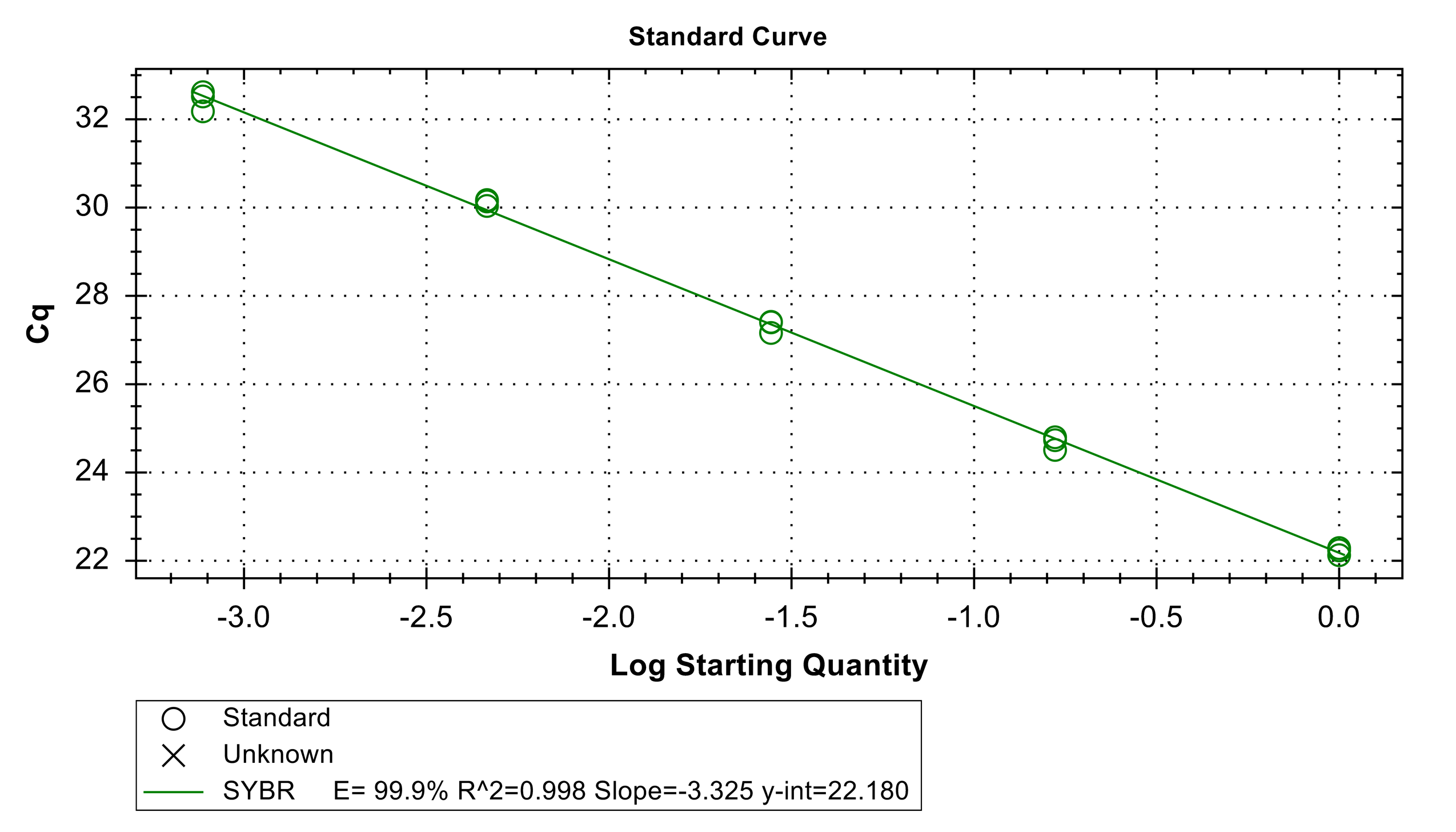

Supplement: Supplementary file 1 [file cimb-44-00288-s001.zip › new-supplementary materials/File folder S1.Standard curves/9336-47.png]

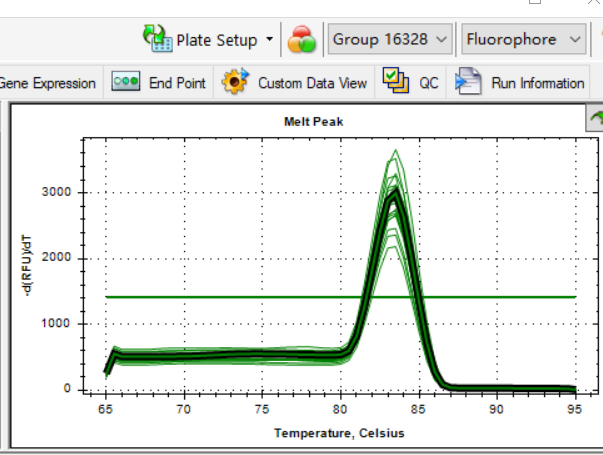

Supplement: Supplementary file 1 [file cimb-44-00288-s001.zip › new-supplementary materials/File folder S2.Melt peak curves/Isoform 16328.png]

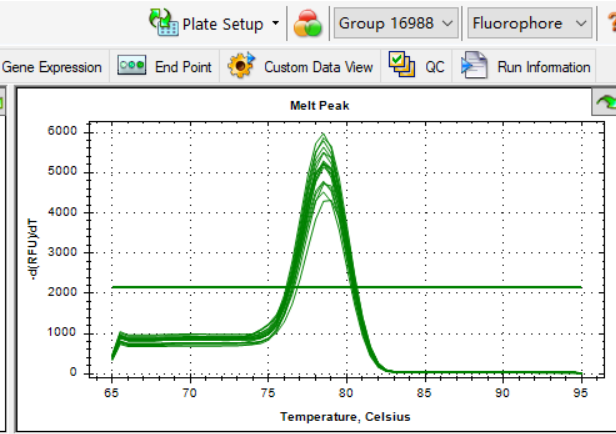

Supplement: Supplementary file 1 [file cimb-44-00288-s001.zip › new-supplementary materials/File folder S2.Melt peak curves/Isoform 16988.png]

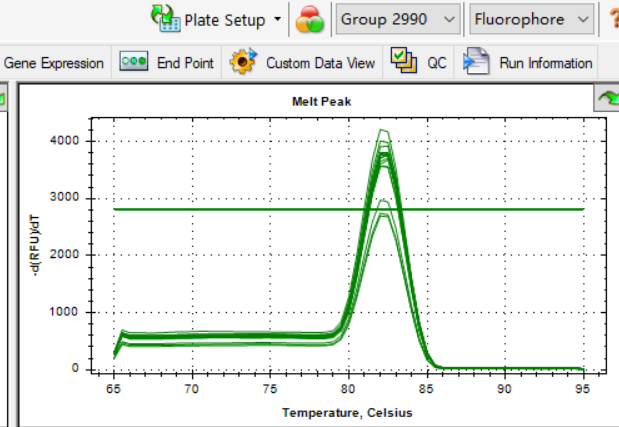

Supplement: Supplementary file 1 [file cimb-44-00288-s001.zip › new-supplementary materials/File folder S2.Melt peak curves/Isoform 2990.png]

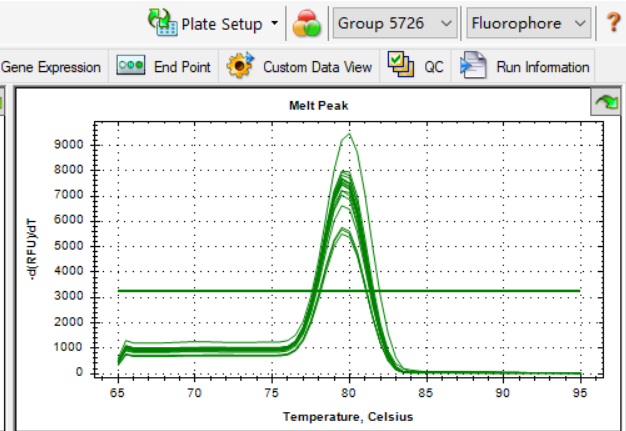

Supplement: Supplementary file 1 [file cimb-44-00288-s001.zip › new-supplementary materials/File folder S2.Melt peak curves/Isoform 5726.png]

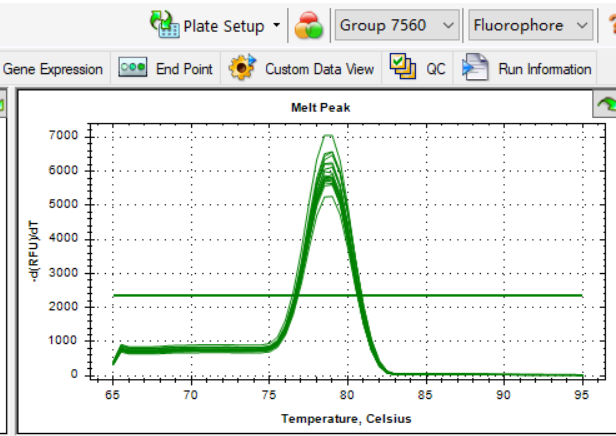

Supplement: Supplementary file 1 [file cimb-44-00288-s001.zip › new-supplementary materials/File folder S2.Melt peak curves/Isoform 7560.png]

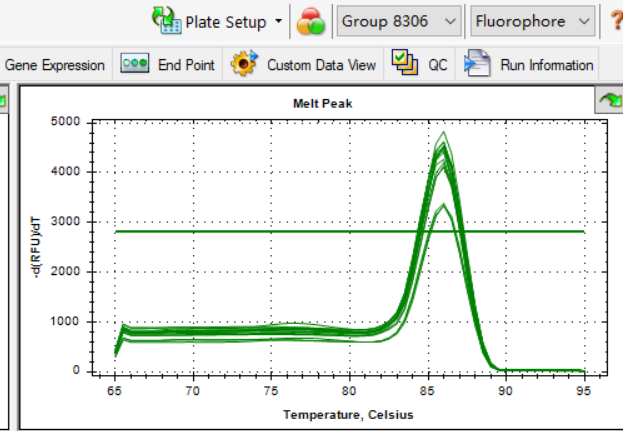

Supplement: Supplementary file 1 [file cimb-44-00288-s001.zip › new-supplementary materials/File folder S2.Melt peak curves/Isoform 8306.png]

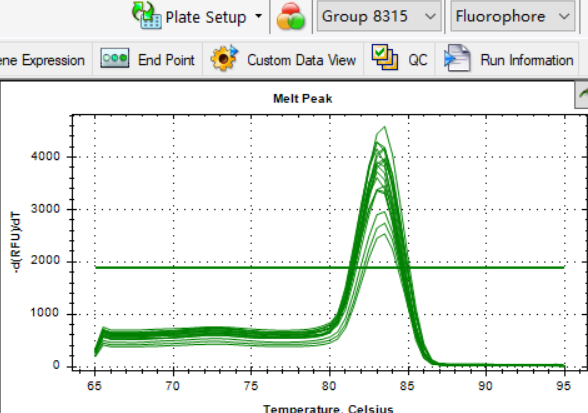

Supplement: Supplementary file 1 [file cimb-44-00288-s001.zip › new-supplementary materials/File folder S2.Melt peak curves/Isoform 8315.png]

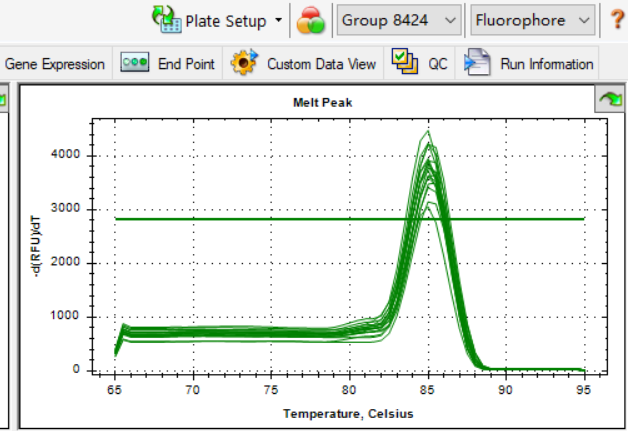

Supplement: Supplementary file 1 [file cimb-44-00288-s001.zip › new-supplementary materials/File folder S2.Melt peak curves/Isoform 8424.png]

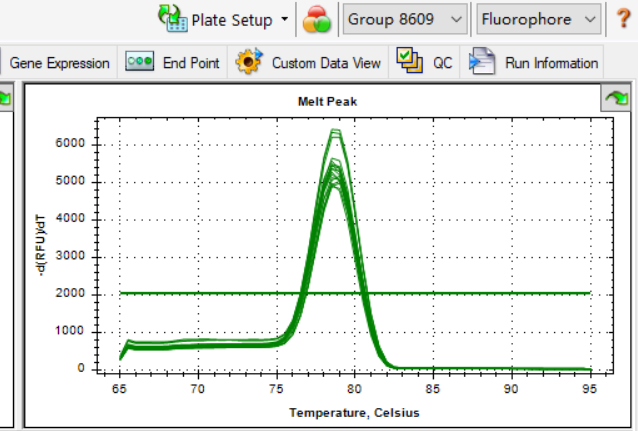

Supplement: Supplementary file 1 [file cimb-44-00288-s001.zip › new-supplementary materials/File folder S2.Melt peak curves/Isoform 8609.png]

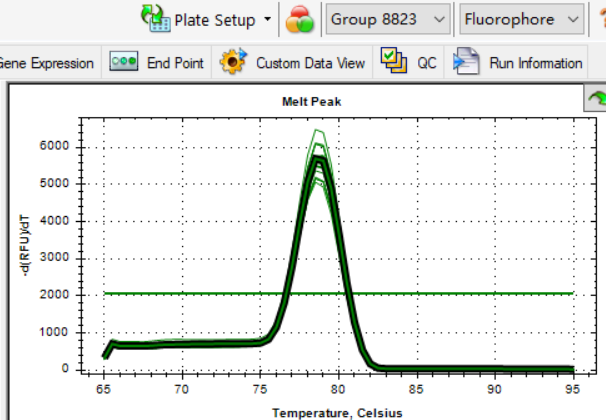

Supplement: Supplementary file 1 [file cimb-44-00288-s001.zip › new-supplementary materials/File folder S2.Melt peak curves/Isoform 8823.png]

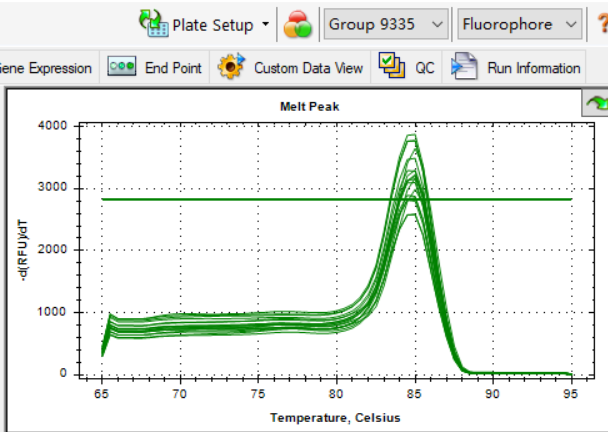

Supplement: Supplementary file 1 [file cimb-44-00288-s001.zip › new-supplementary materials/File folder S2.Melt peak curves/Isoform 9335.png]

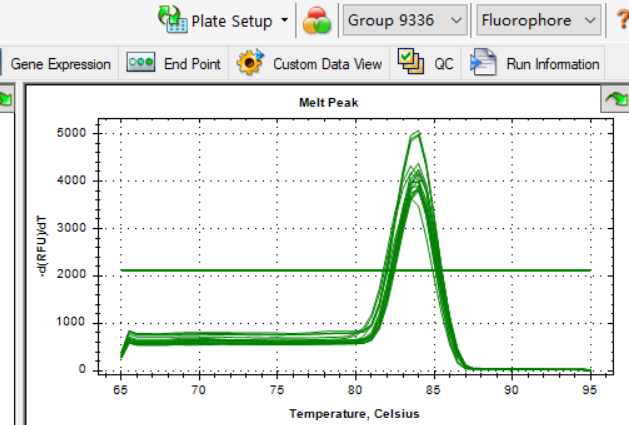

Supplement: Supplementary file 1 [file cimb-44-00288-s001.zip › new-supplementary materials/File folder S2.Melt peak curves/Isoform 9336.png]

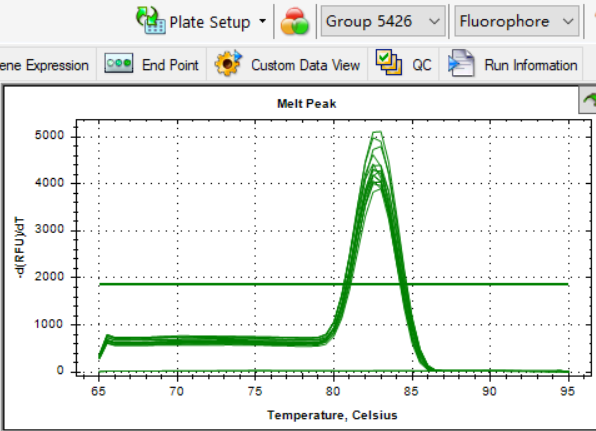

Supplement: Supplementary file 1 [file cimb-44-00288-s001.zip › new-supplementary materials/File folder S2.Melt peak curves/isoform5426.png]

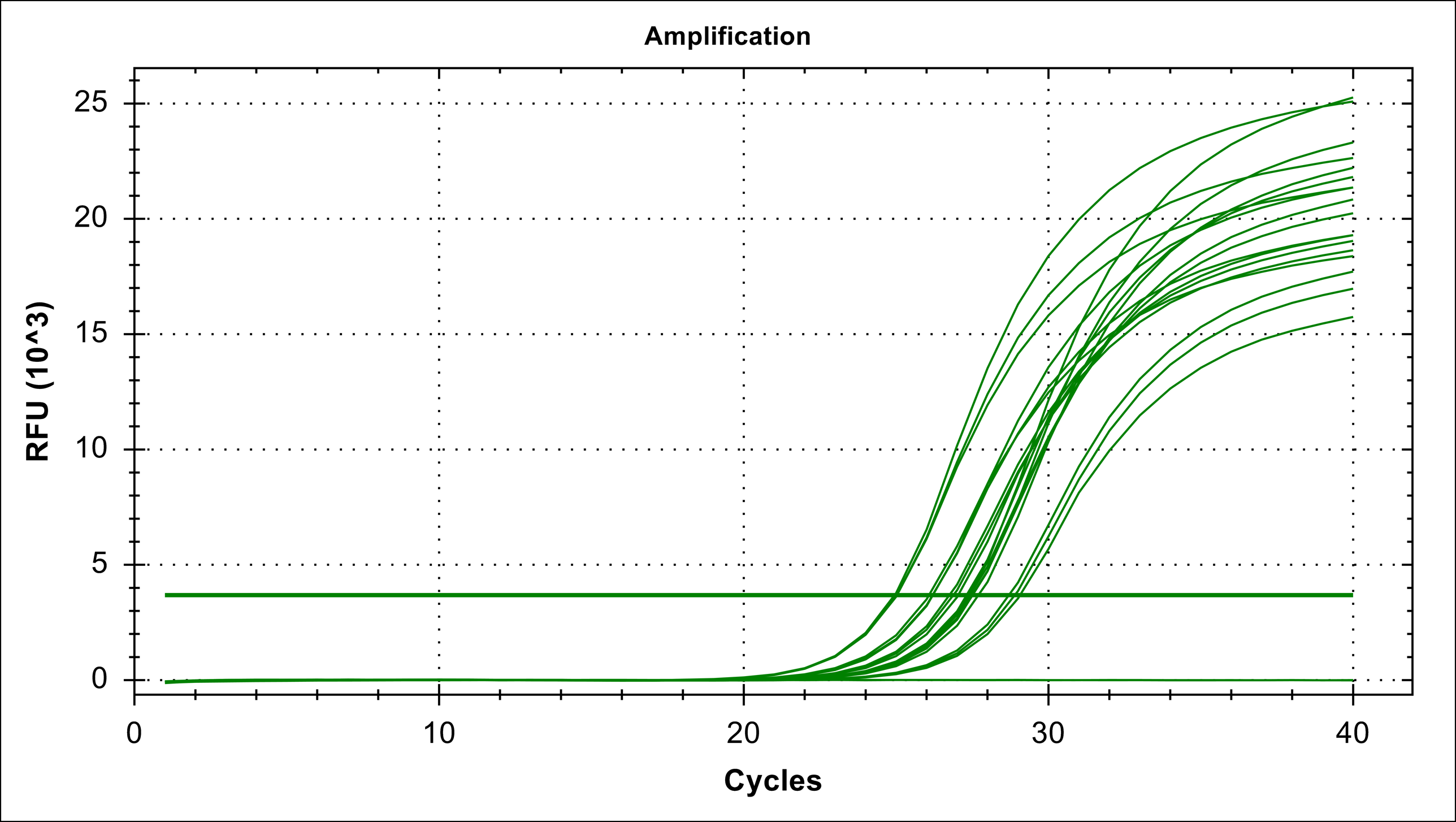

Supplement: Supplementary file 1 [file cimb-44-00288-s001.zip › new-supplementary materials/File folder S3.Amplication curves/Isoform 16328.png]

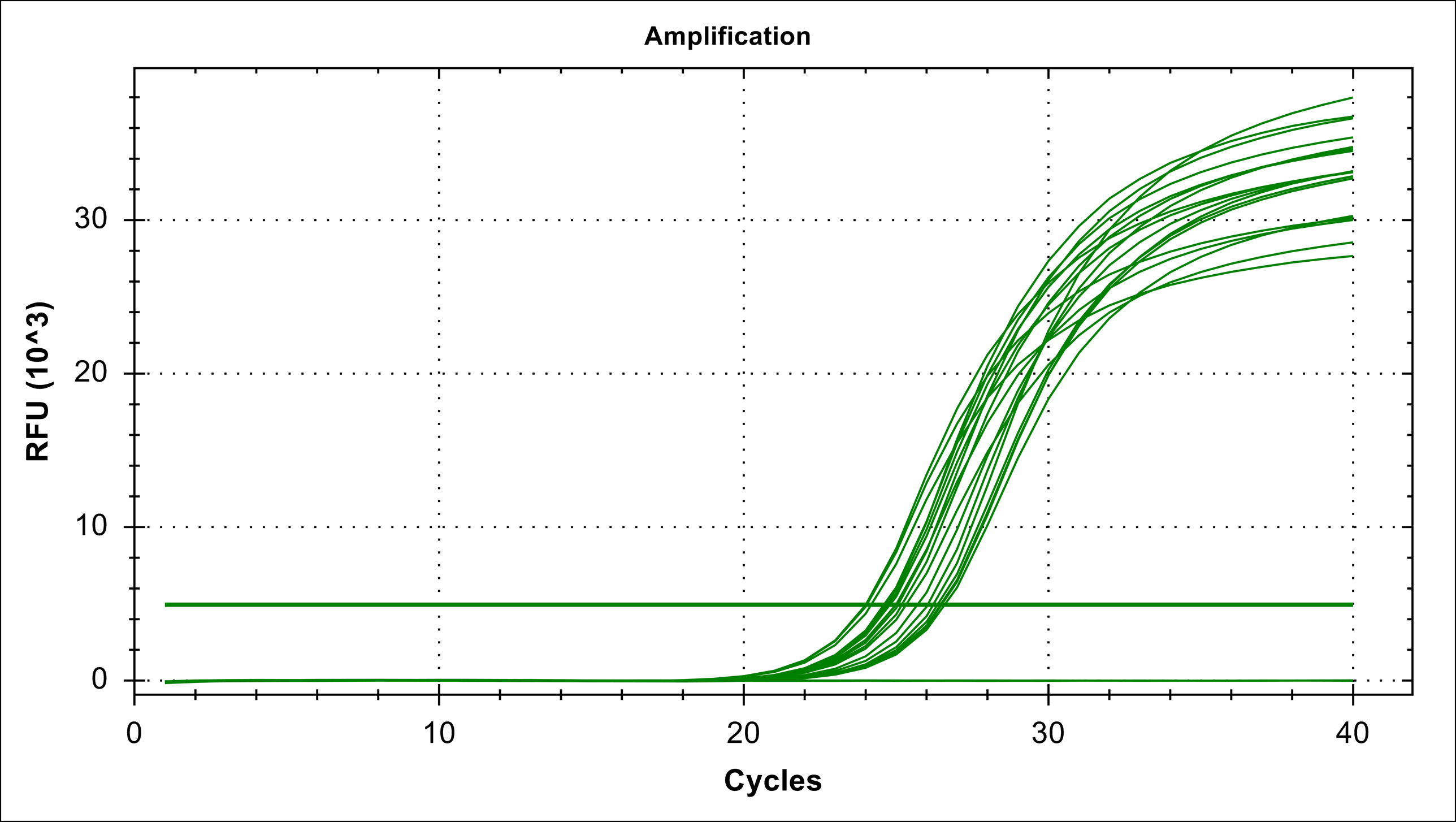

Supplement: Supplementary file 1 [file cimb-44-00288-s001.zip › new-supplementary materials/File folder S3.Amplication curves/Isoform 16988.png]

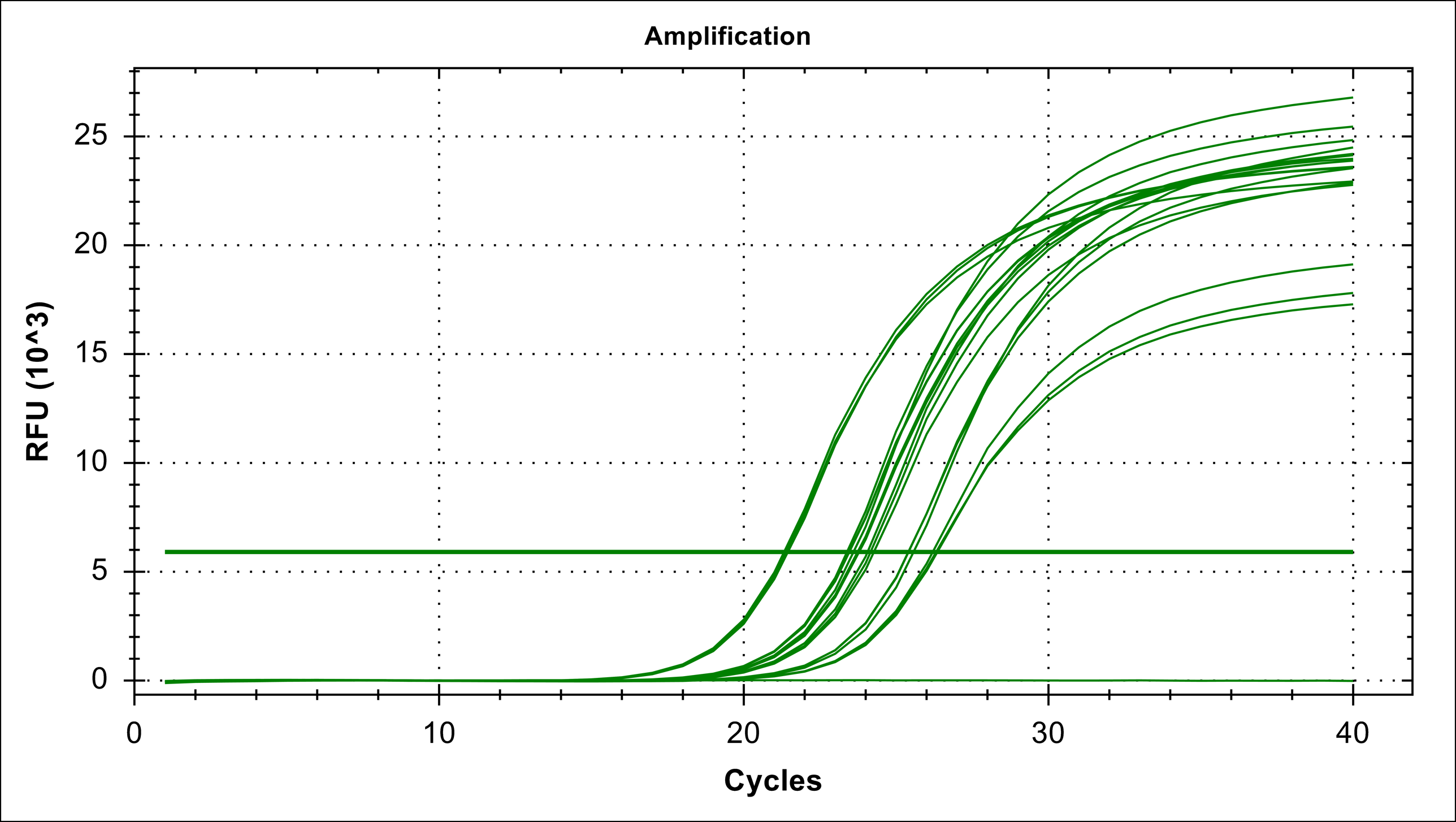

Supplement: Supplementary file 1 [file cimb-44-00288-s001.zip › new-supplementary materials/File folder S3.Amplication curves/Isoform 2990.png]

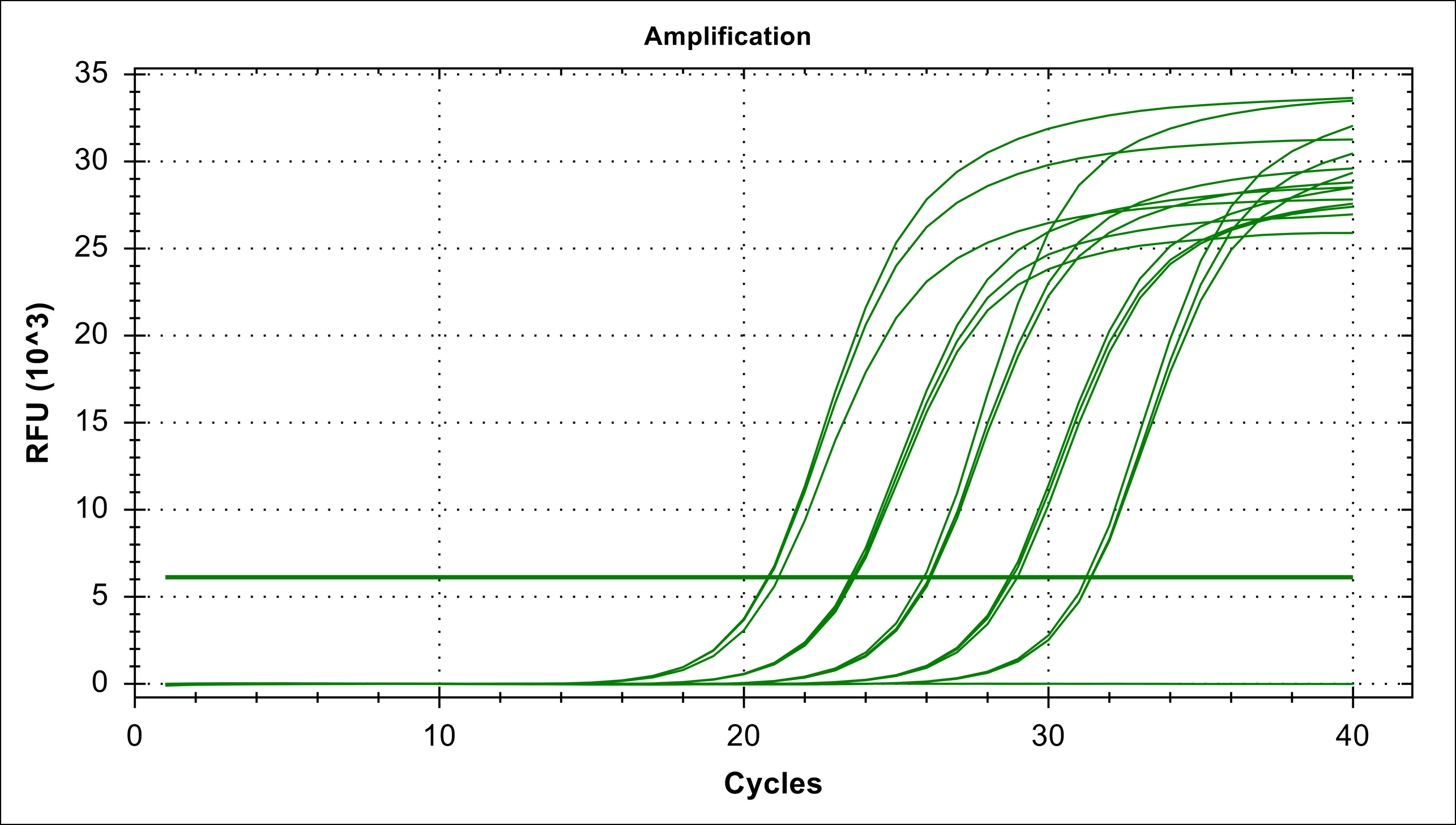

Supplement: Supplementary file 1 [file cimb-44-00288-s001.zip › new-supplementary materials/File folder S3.Amplication curves/Isoform 5426.png]

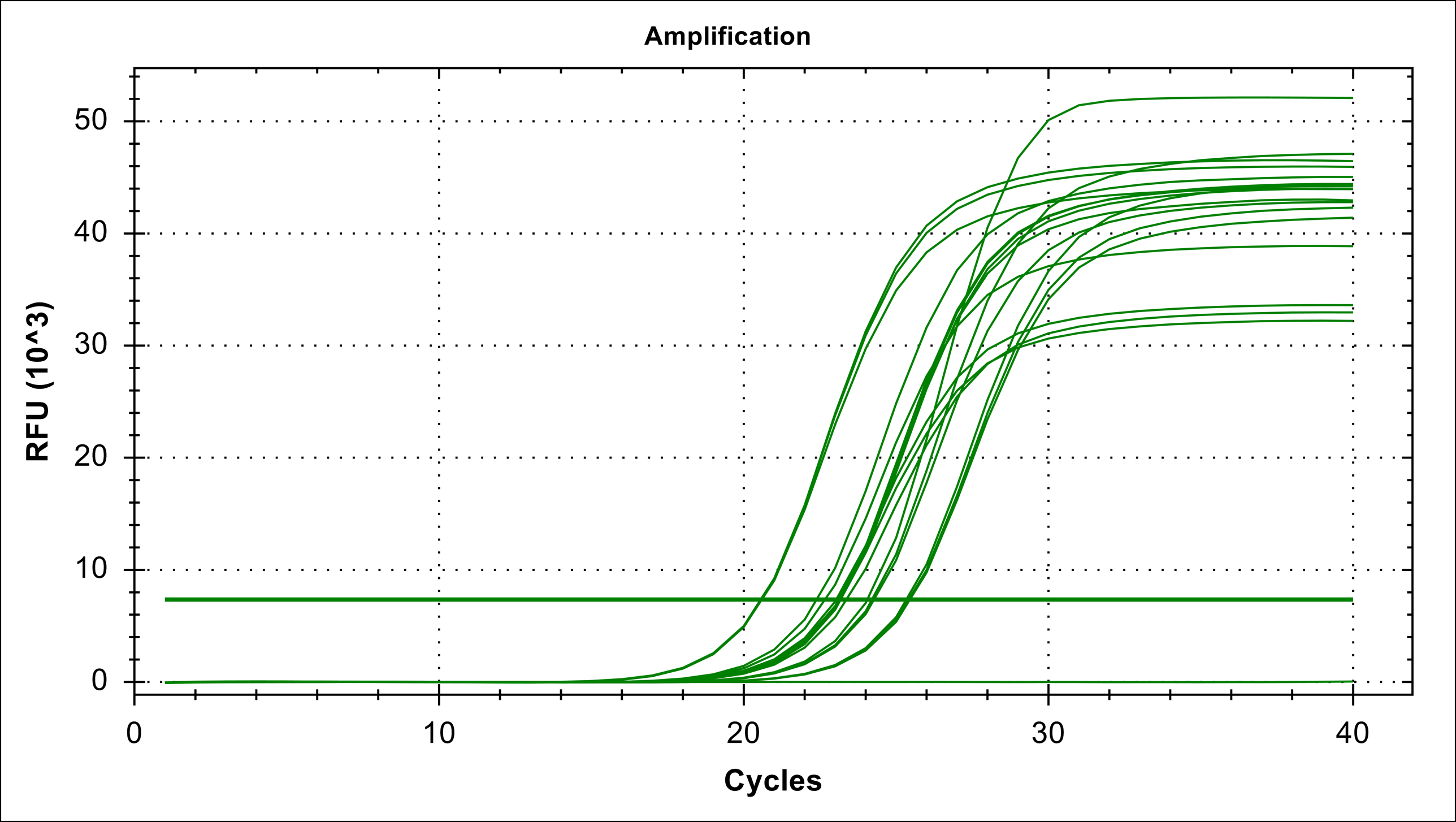

Supplement: Supplementary file 1 [file cimb-44-00288-s001.zip › new-supplementary materials/File folder S3.Amplication curves/Isoform 5726.png]

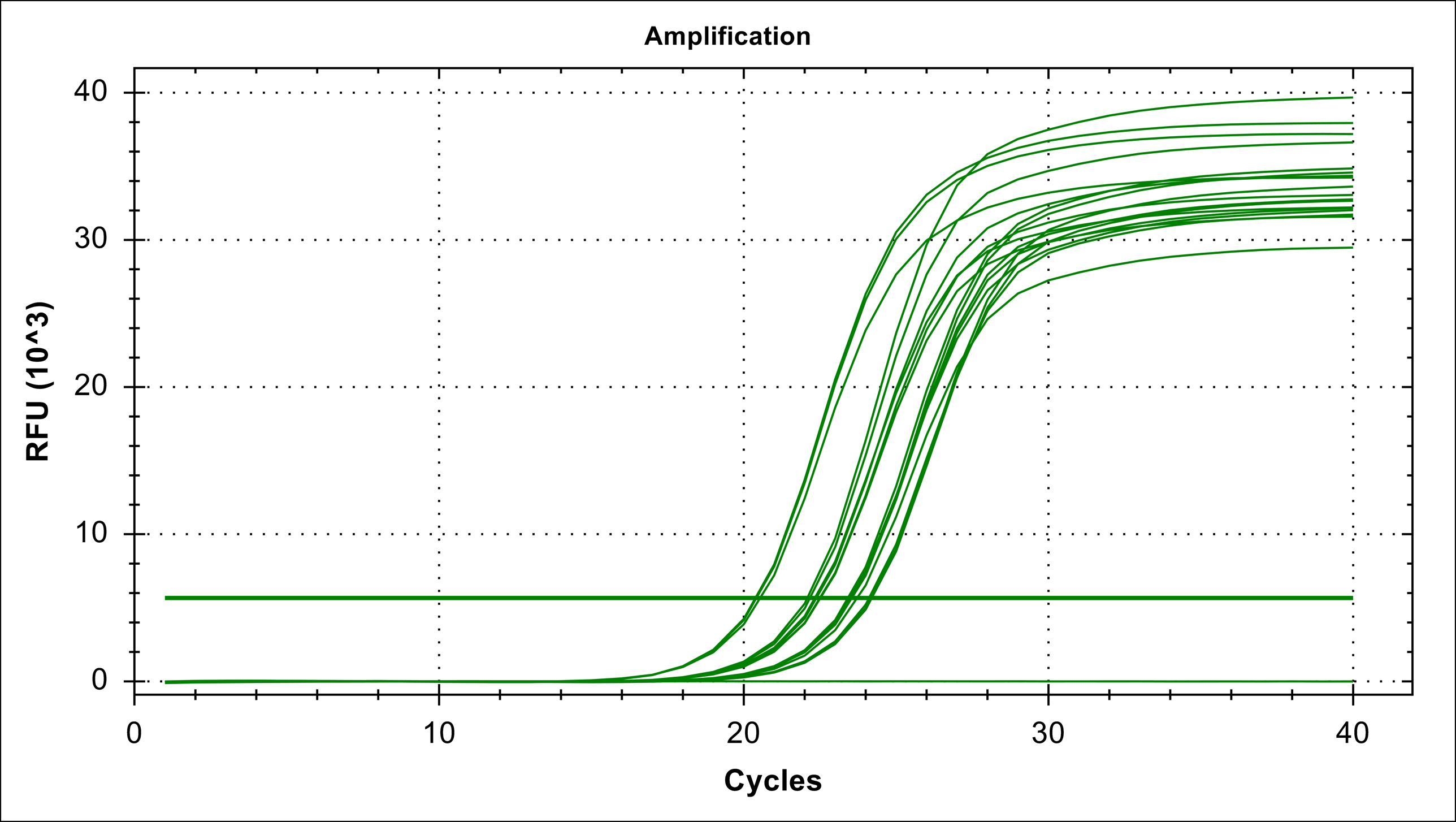

Supplement: Supplementary file 1 [file cimb-44-00288-s001.zip › new-supplementary materials/File folder S3.Amplication curves/Isoform 7506.png]

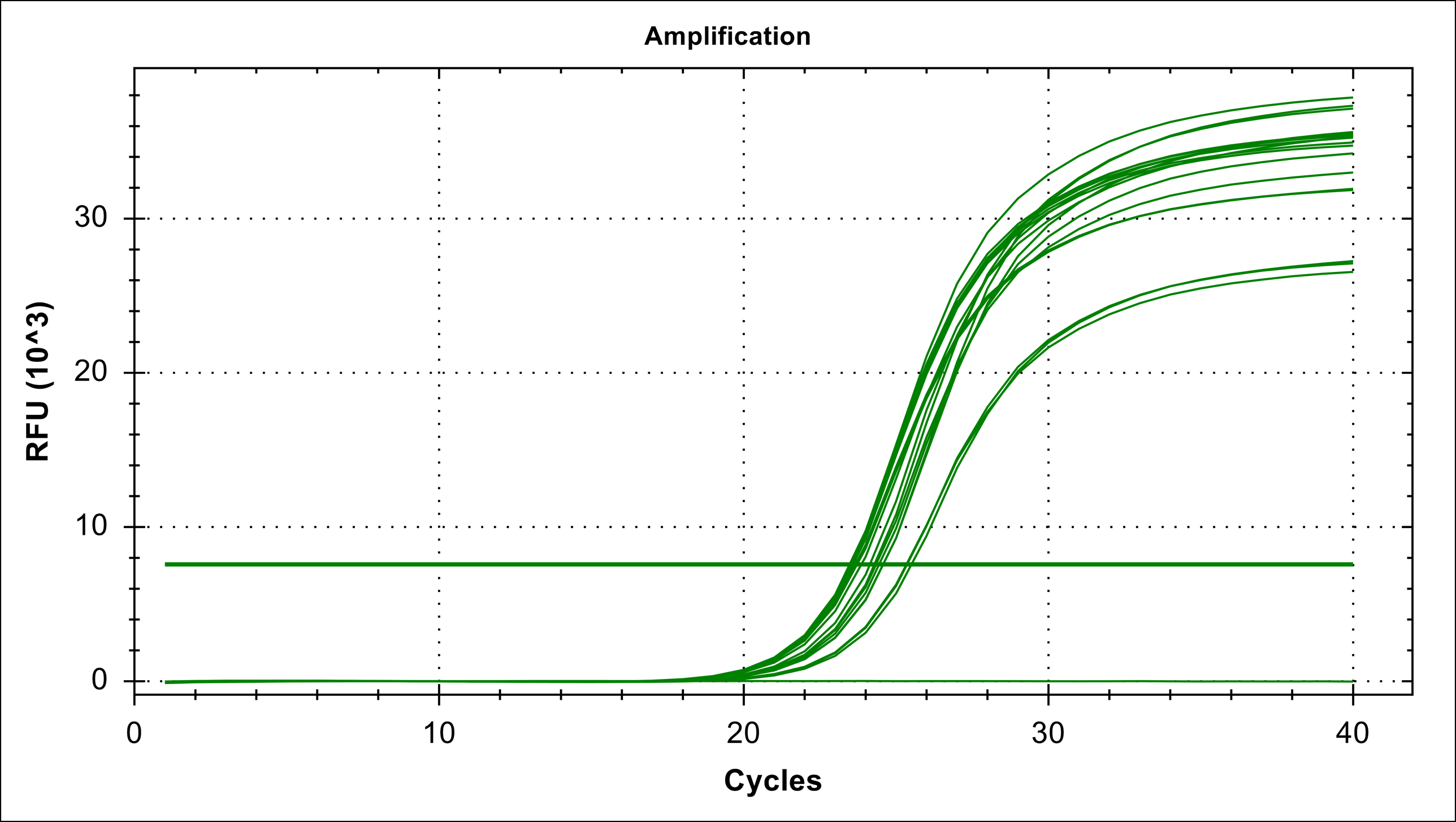

Supplement: Supplementary file 1 [file cimb-44-00288-s001.zip › new-supplementary materials/File folder S3.Amplication curves/Isoform 8306.png]

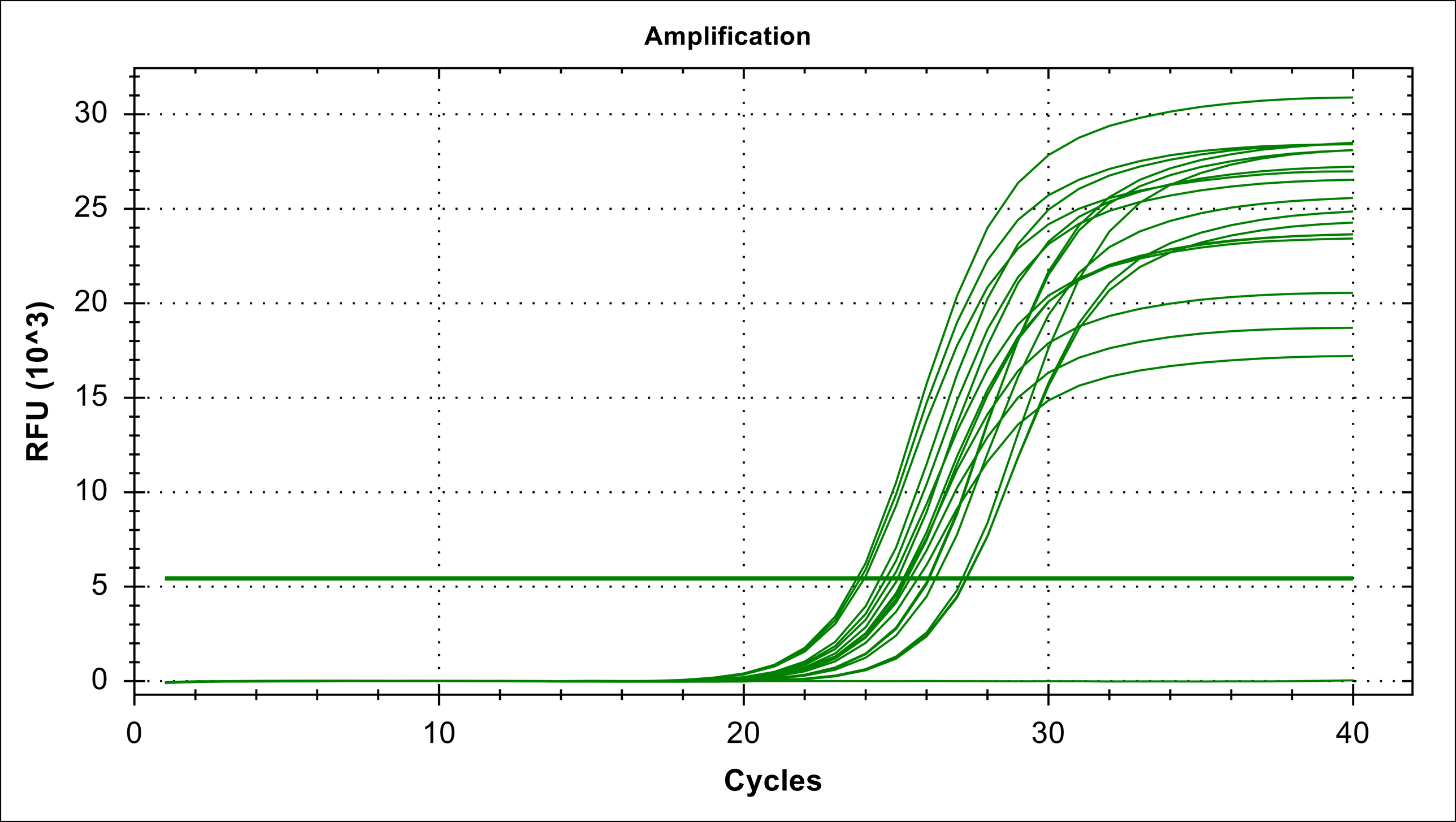

Supplement: Supplementary file 1 [file cimb-44-00288-s001.zip › new-supplementary materials/File folder S3.Amplication curves/Isoform 8315.png]

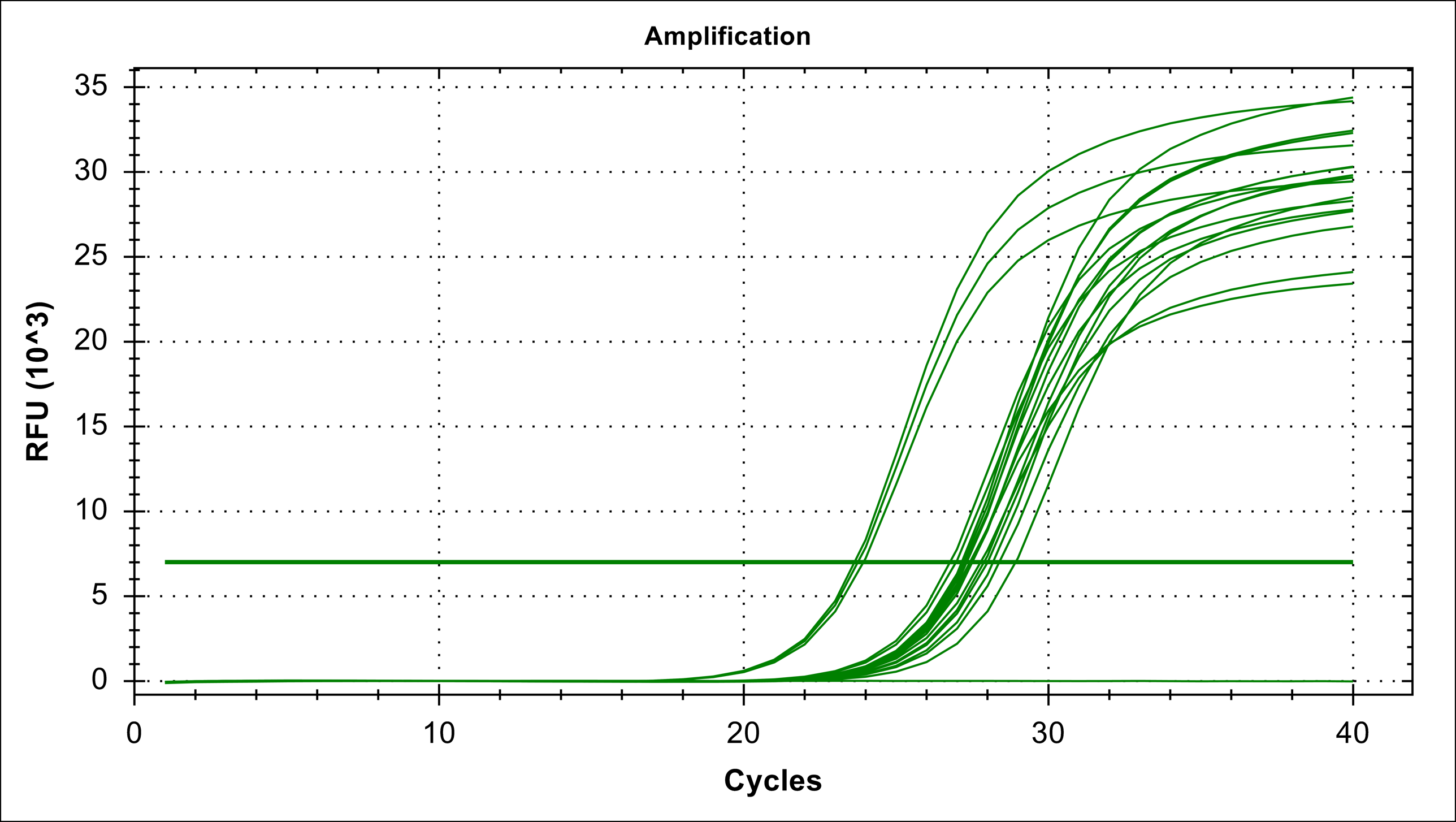

Supplement: Supplementary file 1 [file cimb-44-00288-s001.zip › new-supplementary materials/File folder S3.Amplication curves/Isoform 8424.png]

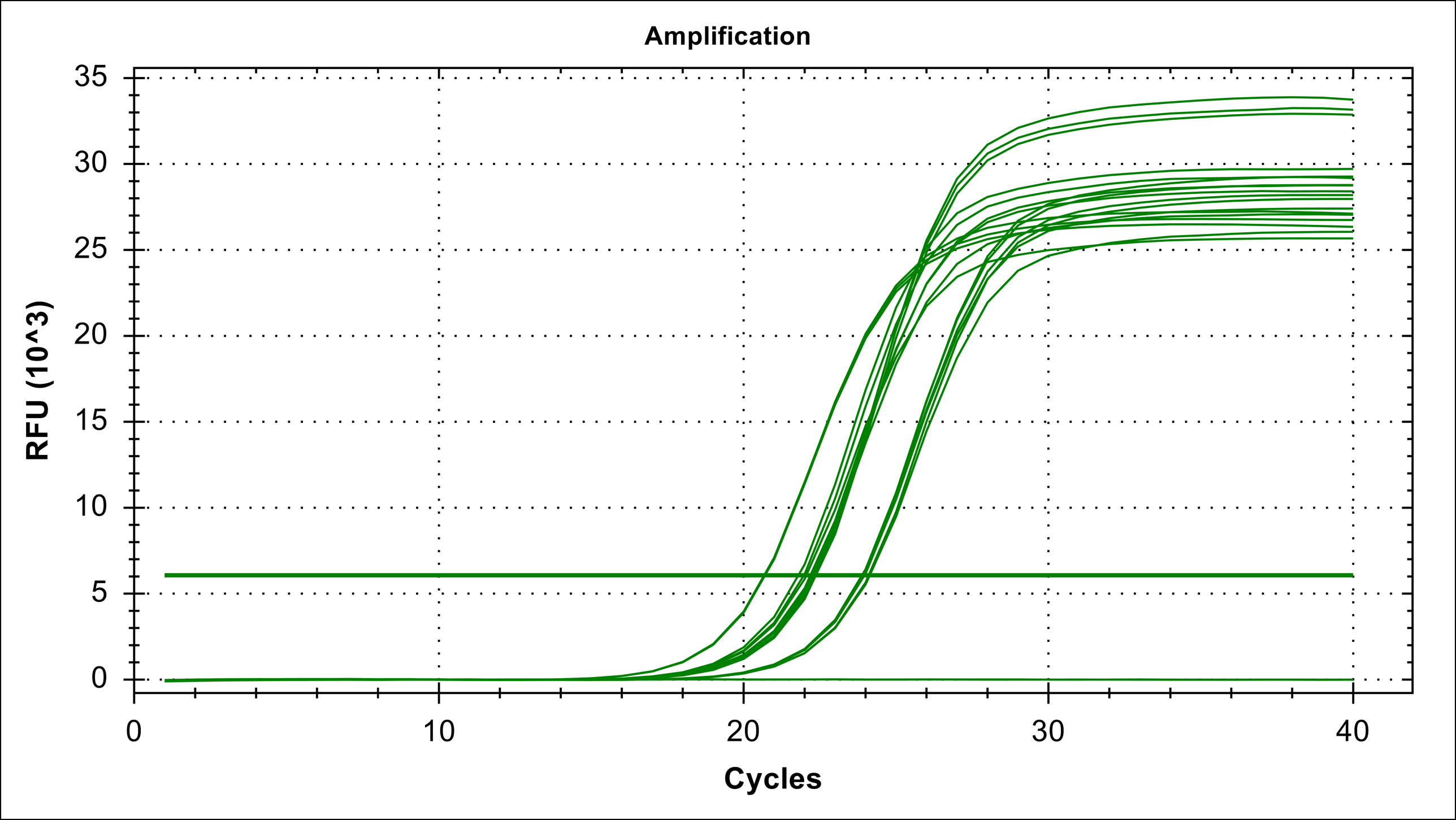

Supplement: Supplementary file 1 [file cimb-44-00288-s001.zip › new-supplementary materials/File folder S3.Amplication curves/Isoform 8609.png]

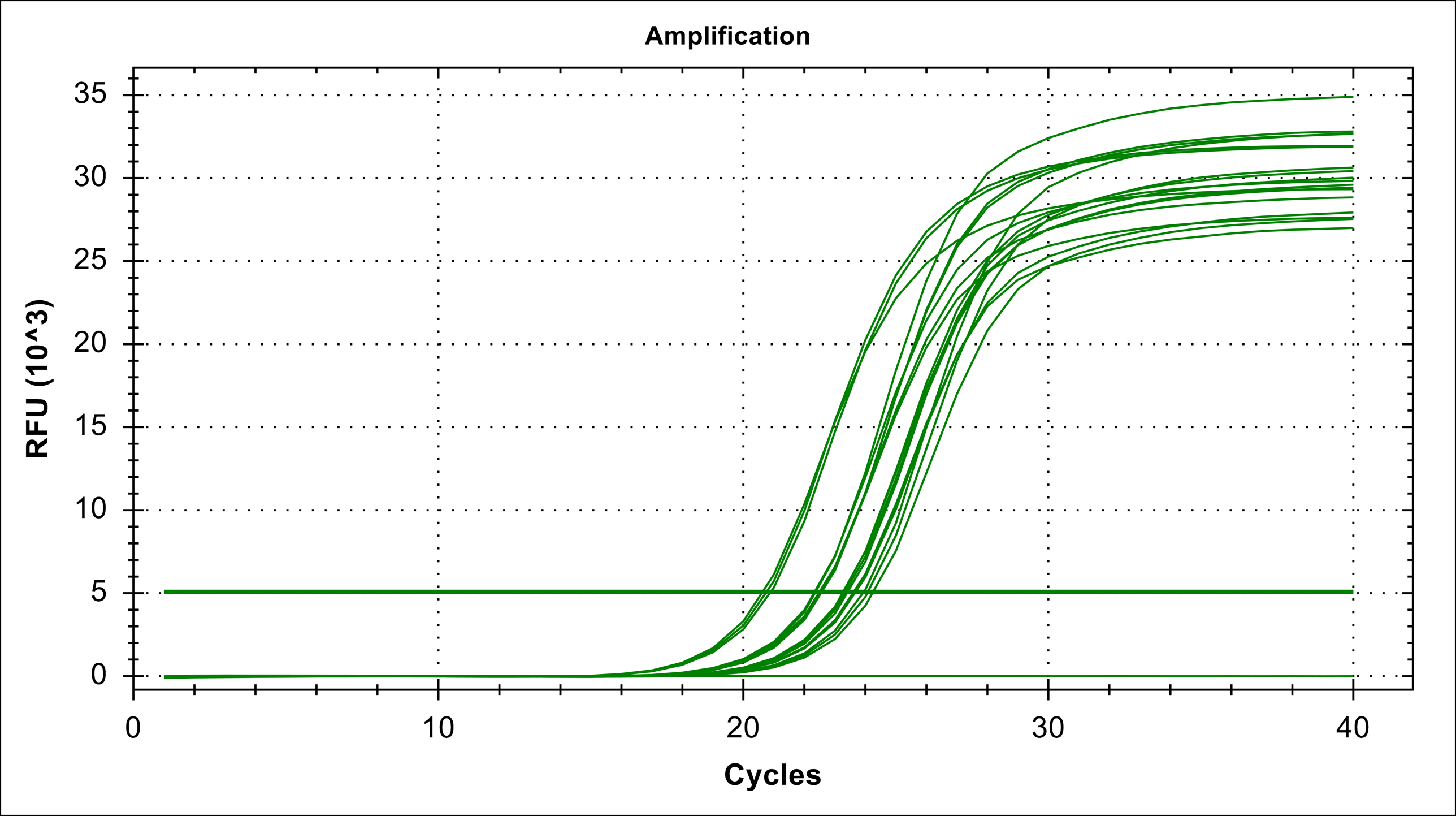

Supplement: Supplementary file 1 [file cimb-44-00288-s001.zip › new-supplementary materials/File folder S3.Amplication curves/Isoform 8823.png]

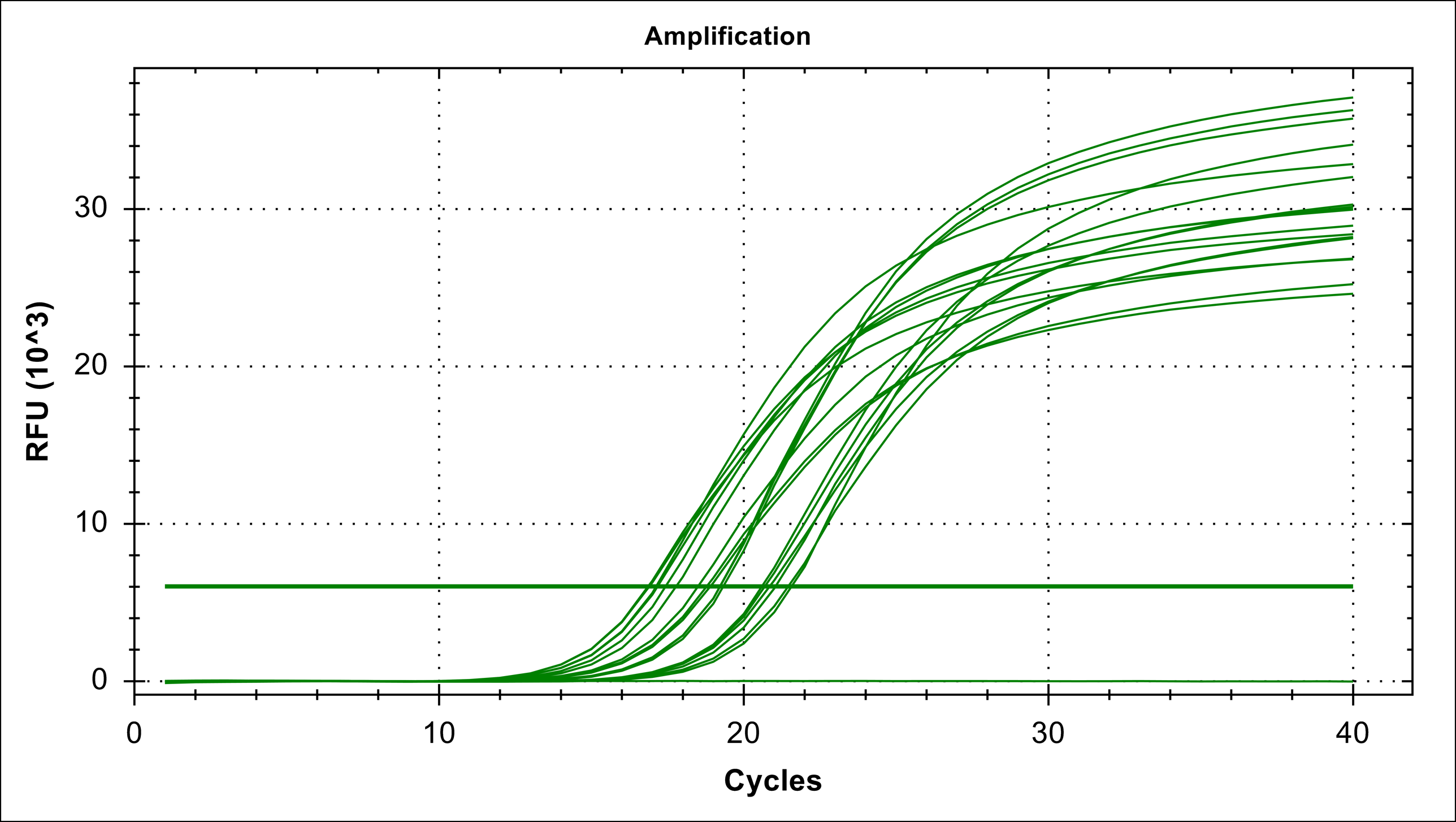

Supplement: Supplementary file 1 [file cimb-44-00288-s001.zip › new-supplementary materials/File folder S3.Amplication curves/Isoform 9335.png]

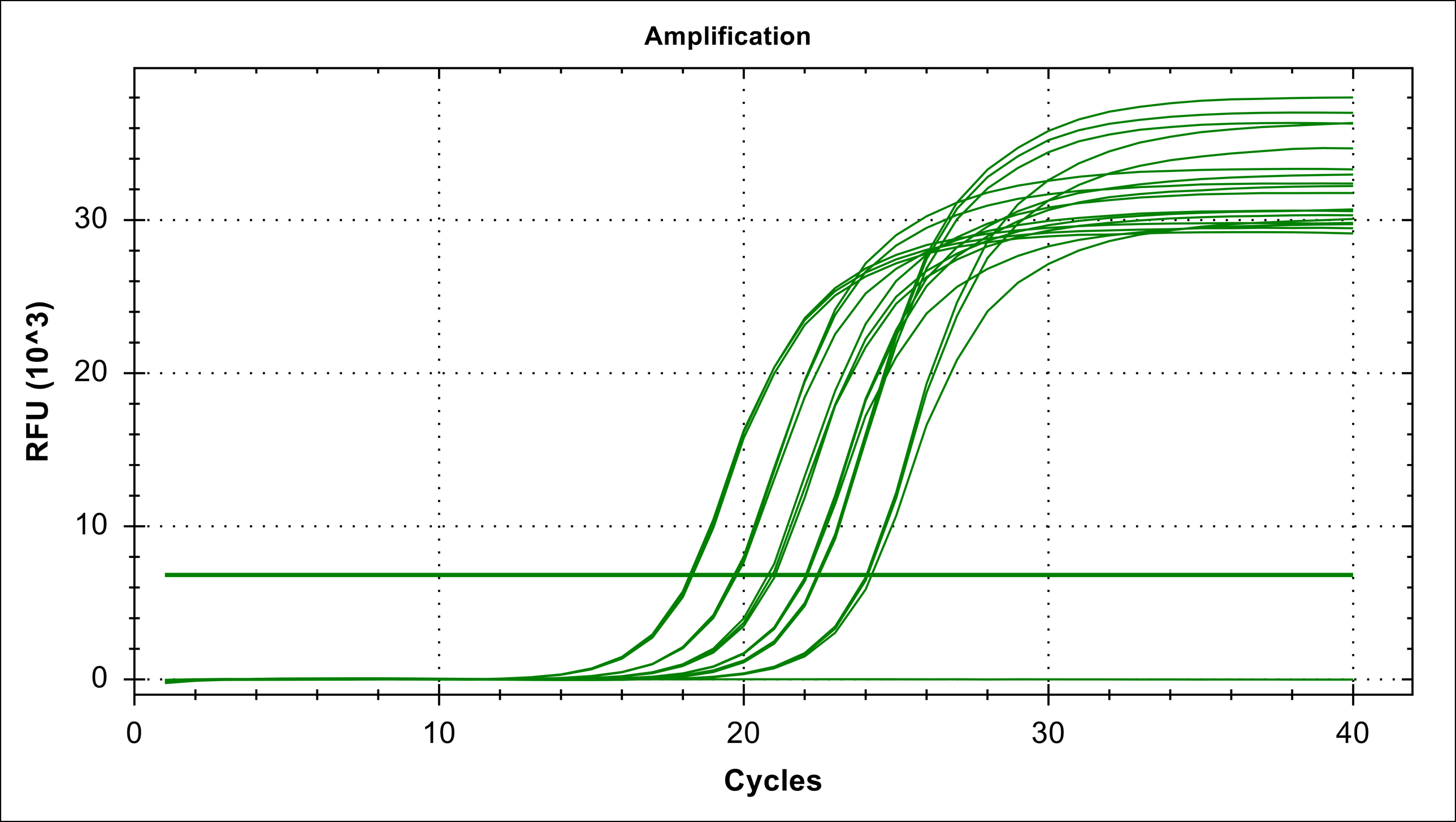

Supplement: Supplementary file 1 [file cimb-44-00288-s001.zip › new-supplementary materials/File folder S3.Amplication curves/Isoform 9336.png]
